# Movement Initiation Signals in Mouse Whisker Motor Cortex

**DOI:** 10.1016/j.neuron.2016.12.001

**Published:** 2016-12-21

**Authors:** Varun Sreenivasan, Vahid Esmaeili, Taro Kiritani, Katia Galan, Sylvain Crochet, Carl C.H. Petersen

**Affiliations:** 1 Laboratory of Sensory Processing, Brain Mind Institute, Faculty of Life Sciences, École Polytechnique Fédérale de Lausanne (EPFL), 1015 Lausanne, Switzerland; 2 Centre for Developmental Neurobiology, King’s College London, London SE1 1UL, UK

**Keywords:** motor cortex, whisker motor control, movement initiation, motor coding, sensorimotor integration, optogenetics, whole-cell recording, membrane potential, multisite silicon probe recording, action potential

## Abstract

Frontal cortex plays a central role in the control of voluntary movements, which are typically guided by sensory input. Here, we investigate the function of mouse whisker primary motor cortex (wM1), a frontal region defined by dense innervation from whisker primary somatosensory cortex (wS1). Optogenetic stimulation of wM1 evokes rhythmic whisker protraction (whisking), whereas optogenetic inactivation of wM1 suppresses initiation of whisking. Whole-cell membrane potential recordings and silicon probe recordings of action potentials reveal layer-specific neuronal activity in wM1 at movement initiation, and encoding of fast and slow parameters of movements during whisking. Interestingly, optogenetic inactivation of wS1 caused hyperpolarization and reduced firing in wM1, together with reduced whisking. Optogenetic stimulation of wS1 drove activity in wM1 with complex dynamics, as well as evoking long-latency, wM1-dependent whisking. Our results advance understanding of a well-defined frontal region and point to an important role for sensory input in controlling motor cortex.

## Highlights

• Optogenetic excitation (inactivation) of wM1 evokes (inhibits) whisking
• Layer-specific neuronal activity in wM1 encodes onset, phase, and envelop of whisking
• Optogenetic inactivation of sensory cortex decreases wM1 activity and whisking
• Optogenetic excitation of sensory cortex initiates whisking dependent upon wM1

## Teaser

Sreenivasan, Esmaeili et al. delineate layer-specific neuronal activity patterns in mouse whisker motor cortex contributing to initiation and control of exploratory whisking. In turn, whisker motor cortex and whisker movements are strongly influenced by neuronal activity in primary somatosensory cortex.

## Introduction

An important goal of neuroscience is to obtain a causal and mechanistic understanding of how voluntary movements are generated by the brain. A key structure that is thought to be involved in the control of movement is motor cortex (Fritsch and Hitzig, 1870, Ferrier, 1874). Seminal work in primates revealed the existence of neuronal populations in motor cortex that encode arm movement onset, movement direction, and precision grip (Evarts, 1968, Georgopoulos et al., 1986). Motor cortex receives axonal innervation from primary somatosensory cortex (Jones et al., 1978), and sensory responses in motor cortex are prominent (Fetz et al., 1980). Synaptic inputs from sensory cortex innervating motor cortex might thus contribute to initiate and guide movements, but the precise nature of such interactions is unknown.

The mouse whisker sensorimotor system provides a relatively simple and well-defined model for investigating motor control and sensorimotor integration. During active exploration, mice typically move their whiskers back and forth at high frequencies (∼10 Hz), scanning the nearby environment. Sensory signals are generated as the whiskers contact objects providing the mouse with spatial and textural information about their surroundings (Petersen, 2007, Diamond et al., 2008). These sensory signals in turn alter whisker movements (Mitchinson et al., 2007, Crochet et al., 2011), presumably to improve acquisition of selected tactile features. On the other hand, if a mouse is at rest, the whiskers are held still. Brief deflection of the whiskers, under such conditions, will typically initiate whisking in some trials (Ferezou et al., 2007, Yamashita et al., 2013), whereas strong prolonged stimulation drives whisker retraction (Matyas et al., 2010). Whisker sensory information therefore plays an important role in the initiation and control of whisker movements, but the underlying neuronal mechanisms are poorly understood.

The whisker primary somatosensory cortex (wS1) and whisker motor cortex (wM1) both appear to contribute directly to whisker motor control (Petersen, 2014). Strong stimulation of wS1 evokes a rapid retraction of the contralateral whiskers, perhaps through its innervation of spinal trigeminal premotor neurons of extrinsic whisker-pad muscles (Matyas et al., 2010, Sreenivasan et al., 2015). Stimulation of wM1 evokes short-latency rhythmic whisker protraction, which appears similar to exploratory whisking. These movements might be driven by wM1 innervation of the facial whisker motor nucleus (Grinevich et al., 2005, Sreenivasan et al., 2015) and brainstem reticular formation, which contains many premotor neurons for whisker protraction (Matyas et al., 2010, Takatoh et al., 2013, Sreenivasan et al., 2015) and a central pattern generator for whisking (Moore et al., 2013, Deschênes et al., 2016). Neurons in frontal cortex have been shown to code various aspects of whisking (Hill et al., 2011, Friedman et al., 2012, Gerdjikov et al., 2013), but the precise layer-specific activity underlying the initiation and control of whisker movements in wM1 has not yet been studied. Indeed, previous studies have shown that animals with frontal cortex lesions including wM1 can still whisk (Welker, 1964, Semba and Komisaruk, 1984), raising the question of the causal role of activity in wM1. Here, using optogenetics we find that wM1 contributes to the initiation of whisking, and, using whole-cell and silicon probe recordings, we delineate the layer-specific pattern of activity at whisker movement initiation. We furthermore find that wS1 inputs to wM1 play an important role, thus contributing to the initiation and control of exploratory whisking.

## Results

### Anterograde Labeling of wS1 Axons in Frontal Cortex

**Figure 1:**
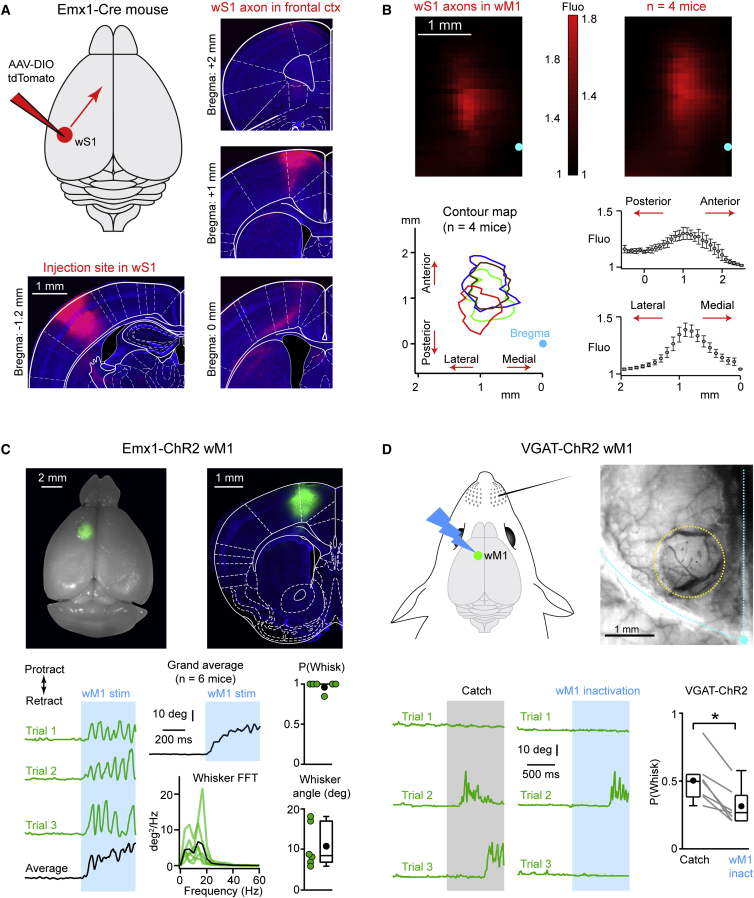
wM1 Plays a Causal Role in Initiation of Exploratory Whisking (A) AAV encoding tdTomato was injected into whisker primary somatosensory cortex (wS1) (left). Serial coronal sections reveal the wS1 pattern of innervation in frontal cortex (right). (B) Example (top left) and grand average (top right) normalized fluorescence intensity map of the wS1 axons in frontal cortex. Contour plots at half-maximum of the normalized fluorescence intensity for four mice (bottom left) show the location of wS1 axons in frontal cortex. Average normalized fluorescence intensity (n = 4 mice) plots across the anterio-posterior and medio-lateral axes (bottom right) show that the wS1 axons peak around 1 mm anterior and 1 mm lateral with respect to bregma. (C) Widefield image of a fixed brain where a conditional ChR2-expressing virus was injected into wM1 (top left). Coronal section showing the injection site localized to wM1 (top right). Three example traces (green) and average trace (black) of the whisker position upon 50 Hz blue light stimulation (bottom left). Grand average trace of the whisker position (black) for six mice upon 50 Hz blue light stimulation of wM1. Only trials without whisking in the prestimulus period were analyzed. Power spectral density of the wM1-driven whisker movement (bottom middle). Green traces are from individual mice and the black trace is the grand average spectrum. The probability of initiating whisker movements, P(Whisk), upon wM1 stimulation is high and the average whisker angle is positive, indicating a protraction (bottom right). Green circles indicate individual mice. Black circle indicates the mean. Boxplots indicate median and interquartile range. (D) Inactivation of wM1 was carried out in VGAT-ChR2 mice (top left). Widefield image showing the surface vasculature and the bone over wM1 (dotted yellow circle) that was thinned prior to inactivation (top right). Bregma (blue circle) and the lateral and midline sutures (blue dotted lines) are also shown. Only trials without whisking in the prestimulus period were analyzed. Three example whisker traces (green) during Catch trials and during wM1 opto-inactivation (bottom left). Note the increased number of failures to initiate whisking during wM1 inactivation. Quantified across animals, the probability to initiate whisking, P(Whisk), was significantly smaller during wM1 inactivation trials compared to Catch trials (bottom right). Gray lines indicate individual mice and black circles indicate mean. Boxplots indicate median and interquartile range. See also Figure S1 and Table S1.

Our first goal was to map the region in frontal cortex that receives axonal input from wS1. To this end, we injected a Cre-dependent adeno-associated virus (AAV) to express tdTomato in wS1 of Emx1-Cre mice, in which Cre-recombinase expression is restricted to excitatory neurons in the neocortex. Following 4 weeks of expression, we cut serial coronal sections of the brains and imaged the axons in frontal cortex as well as the injection site in wS1 (Figure 1A). To delineate the projection site in frontal cortex, we quantified the fluorescence intensity, in a 2 mm window from the midline and extending laterally, in serial sections starting at 0.5 mm posterior to bregma and ending at 2.5 mm anterior to bregma, and normalized these intensity values to the mean background intensity in a region devoid of axons (Figure 1B). Contour analysis of the normalized intensity plots showed that the wS1 innervation in frontal cortex started just frontal to bregma and extended up to 2 mm anterior (Figure 1B). Laterally, wS1 axons formed a narrow band between 0.5 and 1.5 mm (Figure 1B). Analysis of peak location along the anterio-posterior and medio-lateral axes showed that the wS1 innervation in frontal cortex peaked around 1 mm anterior and 1 mm lateral with respect to bregma (anterio-posterior location, 1.15 ± 0.1 mm; medio-lateral location, 0.87 ± 0.04 mm; mean ± sem; n = 4 mice). In line with previous studies, we refer to this anatomically defined region in frontal cortex as wM1 (Ferezou et al., 2007, Aronoff et al., 2010).

### Optogenetic Stimulation of wM1 Evokes Whisking

Having identified the location of wM1, we next investigated the role of this frontal region in controlling whisker movement. In order to optogenetically stimulate excitatory neurons, we injected Cre-dependent AAV to express ChR2 in wM1 of Emx1-Cre mice (Figure 1C; Table S1, available online). Following 4 weeks of expression, we applied a 0.5 s blue light train of 50 Hz to wM1 of awake head-restrained mice and filmed whisker movements at 500 Hz (Figure 1C). We selected trials in which prestimulus whisking was absent. Stimulation of wM1 drove rhythmic protraction of the contralateral C2 whisker at short latencies (median change in whisker angle = 8.5 deg; median 5–20 Hz power = 43 deg^2^; median latency = 25 ms; n = 6 mice) (Figure 1C). Furthermore, the probability of initiating movement upon stimulation was high (median = 1.0; n = 6 mice). These data are consistent with previously published results showing that wM1 stimulation drives rhythmic protraction of the contralateral whiskers that resembles exploratory whisking (Matyas et al., 2010, Sreenivasan et al., 2015).

### Optogenetic Inhibition of wM1 Reduces Spontaneous Whisking

We next optogenetically inactivated wM1 in order to test if spontaneous whisking depended upon wM1 activity. In VGAT-ChR2 mice, a 1 s blue light flash was applied over the thinned bone covering wM1 (Figure 1D; Table S1). In these mice, local photo-activation of ChR2-expressing GABAergic neurons suppresses activity in nearby pyramidal cells (Guo et al., 2014). Trials were only included in the analysis if the mouse was not whisking in the prestimulus period. In order to determine the probability of spontaneous whisking, “Catch” trials were randomly interspersed with “Opto-inactivation” trials. Unilateral stimulation of the ChR2-expressing GABAergic neurons in wM1 led to a significant drop in the probability of initiating whisking (median whisk probability, Catch trials = 0.49 versus wM1 Opto-inactivation trials = 0.26; n = 7 mice; Wilcoxon signed-rank test, p = 0.015) (Figure 1D). As a control, we used GAD67-GFP mice, finding no difference in the probability of initiating whisking comparing “Light-on” and Catch trials (median whisk probability, Catch trials = 0.47 versus Light-on trials = 0.51; n = 7 mice; Wilcoxon signed-rank test, p = 0.56). These results suggest that activity in wM1 plays an important role in initiating bouts of exploratory whisking, contributing to driving approximately 45% of self-initiated whisking bouts under our experimental conditions.

### Spontaneous Movement Initiation Signals in wM1

**Figure 2:**
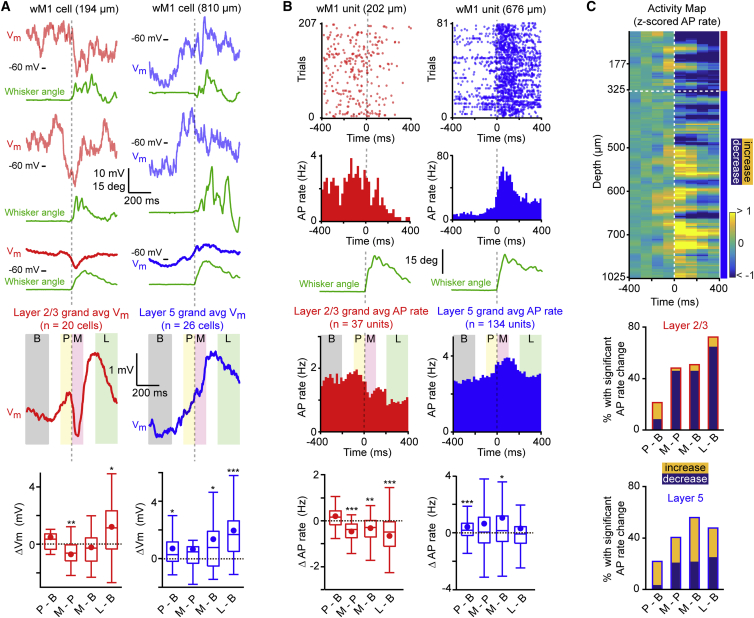
Membrane Potential and AP Dynamics in wM1 during Whisker Movement Initiation (A) Example V_m_ recordings from L2/3 (red) and L5 (blue) neurons aligned to whisker movement onset (green) (top). Lighter V_m_ traces indicate single trials and darker traces indicate mean. Note the pronounced V_m_ hyperpolarization in the L2/3 neuron near movement onset and the V_m_ depolarization in the L5 neuron before movement onset. Grand average V_m_ traces for L2/3 (red) and L5 (blue) aligned to whisker movement onset (middle). Four epochs of interest are delineated (B, Baseline; P, Pre-movement; M, Movement-onset; L, Late during ongoing whisking). Changes in membrane potential (ΔV_m_) quantified across the different epochs (bottom). On average, L2/3 neurons hyperpolarized significantly during Movement-onset, but depolarized significantly during the Late period. L5 neurons depolarized significantly during the Pre-movement period and remained depolarized during Movement-onset and Late periods. Circles indicate mean. Boxplots indicate median and interquartile range. (B) Raster plots and corresponding peri-stimulus time histograms (PSTHs) for L2/3 (red) and L5 (blue) units, aligned to whisker movement onset (green) (top). Grand average AP rates for L2/3 and L5 aligned to whisker movement onset (middle). Changes in AP rate (ΔAP rate) quantified across the different epochs (bottom). On average, L2/3 units significantly reduced AP firing rates during the Movement-onset and Late periods. L5 units significantly increased AP firing rates during the Pre-movement and Movement-onset periods but returned to Baseline during the Late period. Circles indicate mean. Boxplots indicate median and interquartile range. (C) Laminar map of spiking activity (top). The z-scored PSTHs of individual units (100 ms bin size) were aligned to whisking onset and sorted according to their depth. A smoothing window (with size of 5 units) was applied across depth to obtain the smooth activity map. Note the distinct activity patterns in L2/3 and L5. Percentage of wM1 units with significant changes in AP rate for L2/3 (middle) and L5 (bottom) across different epochs. Blue and yellow coloring indicates significant decrease and increase in AP rates, respectively. See also Figure S2 and Table S2.

wM1 thus appears to causally participate in initiating exploratory whisking. It is therefore of interest to investigate the dynamics of the neuronal signals in wM1 at whisker movement onset. We thus made in vivo whole-cell patch-clamp recordings of membrane potential (V_m_), and multisite silicon probe extracellular recordings of action potential (AP) firing in wM1 of awake head-restrained mice (Figure S1), while simultaneously filming movements of the contralateral C2 whisker. Whole-cell recordings (n = 46 cells in N = 38 mice) were obtained from excitatory neurons located between 150 and 850 μm below the pia. Silicon probe recordings (n = 171 putative excitatory units in N = 5 mice) were obtained from neurons between 50 and 1,025 μm below the pia. Single units in wM1 were categorized as putative excitatory or inhibitory neurons based on the duration of the spike waveform, and, in this study, we specifically focus on the putative excitatory units (Figure S1). We further classified neurons as belonging to either layer 2/3 (L2/3) or layer 5 (L5) based upon layer boundaries determined in Etv1-CreERT2 × LSL-tdTomato mice (Figure S1). We aligned individual V_m_ traces and spike time histograms to movement onset, and analyzed four time periods around whisking initiation: (1) “Baseline” from −400 to −200 ms, (2) “Pre-movement” from −100 to 0 ms, (3) “Movement-onset” from 0 to +100 ms, and (4) “Late” during ongoing whisking from +200 to +400 ms. We found striking differences in V_m_ dynamics and spiking activity between L2/3 and L5 neurons across these different phases (Figure 2; Table S2).

Whole-cell recordings revealed that the mean V_m_ of neurons in L2/3 was significantly hyperpolarized relative to L5 neurons in the Baseline period before whisking onset (median V_m_; L2/3 = −53.1 mV, n = 20 cells; L5 = −49.4 mV, n = 26 cells; Wilcoxon-Mann-Whitney test, p = 0.04), while AP thresholds were not significantly different (median AP threshold; L2/3 = −33.8 mV, n = 14 cells; L5 = −35.0 mV, n = 19 cells; Wilcoxon-Mann-Whitney test, p = 0.97). Consistent with L5 neurons being more depolarized and closer to AP threshold, silicon probe recordings showed that the Baseline AP rate of putative excitatory units in L5 was significantly higher than in L2/3 (median AP rate; L2/3 = 1.25 Hz, n = 37 units; L5 = 1.72 Hz, n = 134 units; Wilcoxon-Mann-Whitney test, p = 0.03).

L5 neurons depolarized significantly during the Pre-movement phase relative to Baseline (median ΔV_m_ = 0.26 mV, n = 26 cells; Wilcoxon signed-rank test, p = 0.02) (Figure 2A). Extracellular unit recordings showed that this depolarization was accompanied by a significant increase in Pre-movement AP rates in L5 (median ΔAP rate = 0.18 Hz, n = 134 units; Wilcoxon signed-rank test, p = 6 × 10^−4^) (Figure 2B); 19.4% of L5 units significantly increased firing rate and only 3.7% showed a significant decrease (Figure 2C). Although there was a similar trend in L2/3 (median ΔV_m_ = 0.35 mV, n = 20 cells; 10.8% of units increasing and 8.1% of units decreasing firing rate significantly), the change in V_m_ and AP rates during Pre-movement relative to Baseline did not reach statistical significance across the population (Figures 2A–2C).

During the Movement-onset phase, L5 cells continued to remain depolarized relative to the Baseline period (median ΔV_m_ = 0.77 mV, n = 26 cells; Wilcoxon signed-rank test, p = 0.01) (Figure 2A). AP firing rates in L5 also remained elevated during Movement-onset compared to Baseline (median ΔAP rate = 0.15 Hz, n = 134 units; Wilcoxon signed-rank test, p = 0.03) (Figure 2B); 32.1% of L5 units significantly increased firing rate and 22.4% showed a decrease (Figure 2C). On the other hand, L2/3 cells showed a sharp hyperpolarization at the Movement-onset compared to Pre-movement period (median ΔV_m_ = −0.62 mV, n = 20 cells; Wilcoxon signed-rank test, p = 0.01) (Figure 2A) along with a significant drop in AP rate (median ΔAP rate = −0.38 Hz, n = 37 units; Wilcoxon signed-rank test, p = 4 × 10^−5^) (Figure 2B); 48.7% of L2/3 units significantly decreased firing rate and only 2.7% showed a significant increase (Figure 2C).

Finally, during the Late phase with ongoing whisking, both L2/3 cells (median ΔV_m_ = 1.1 mV, n = 20 cells; Wilcoxon signed-rank test, p = 0.02) and L5 cells (median ΔV_m_ = 1.7 mV, n = 26 cells; Wilcoxon signed-rank test, p = 4 × 10^−5^) were significantly depolarized compared to Baseline (Figure 2A). Surprisingly, we did not observe an increase in AP rate for either population during the Late whisking period compared to Baseline. On the contrary, AP firing rates in L2/3 remained suppressed (median ΔAP rate = −0.50 Hz, n = 37 units; Wilcoxon signed-rank test, p = 0.0002) (Figure 2B); 67.6% of units significantly reduced AP rates and only 8.1% increased firing rate (Figure 2C). AP firing in L5 units on average remained unchanged with respect to baseline (median ΔAP rate = −0.08 Hz, n = 134 units; Wilcoxon signed-rank test, p = 0.40) (Figure 2B); 18.7% of units significantly increased and 26.9% significantly decreased firing rate (Figure 2C). That L2/3 and L5 neurons depolarize during whisking but do not increase firing rate may, at least in part, be due to the dependence of AP rates on not only the mean V_m_ but also on the SD of the V_m_ fluctuations (Figure S2); with APs in excitatory cortical neurons generally being driven by large rapid pre-spike depolarizations (Poulet and Petersen, 2008, Gentet et al., 2010).

Overall, L2/3 and L5 cells in wM1 thus displayed distinct V_m_ dynamics and spiking before whisker movement onset and during whisking. L2/3 neurons rapidly hyperpolarized and reduced AP firing at whisker movement onset, whereas L5 neurons depolarized and increased firing in Pre-movement and Movement-onset phases, perhaps acting as a motor command to initiate whisking. During ongoing whisking, two-thirds of L2/3 neurons remained suppressed, and almost half of L5 neurons were significantly modulated without an overall change in population firing rate.

### V_m_ and APs in wM1 Encode Whisker Motion

**Figure 3:**
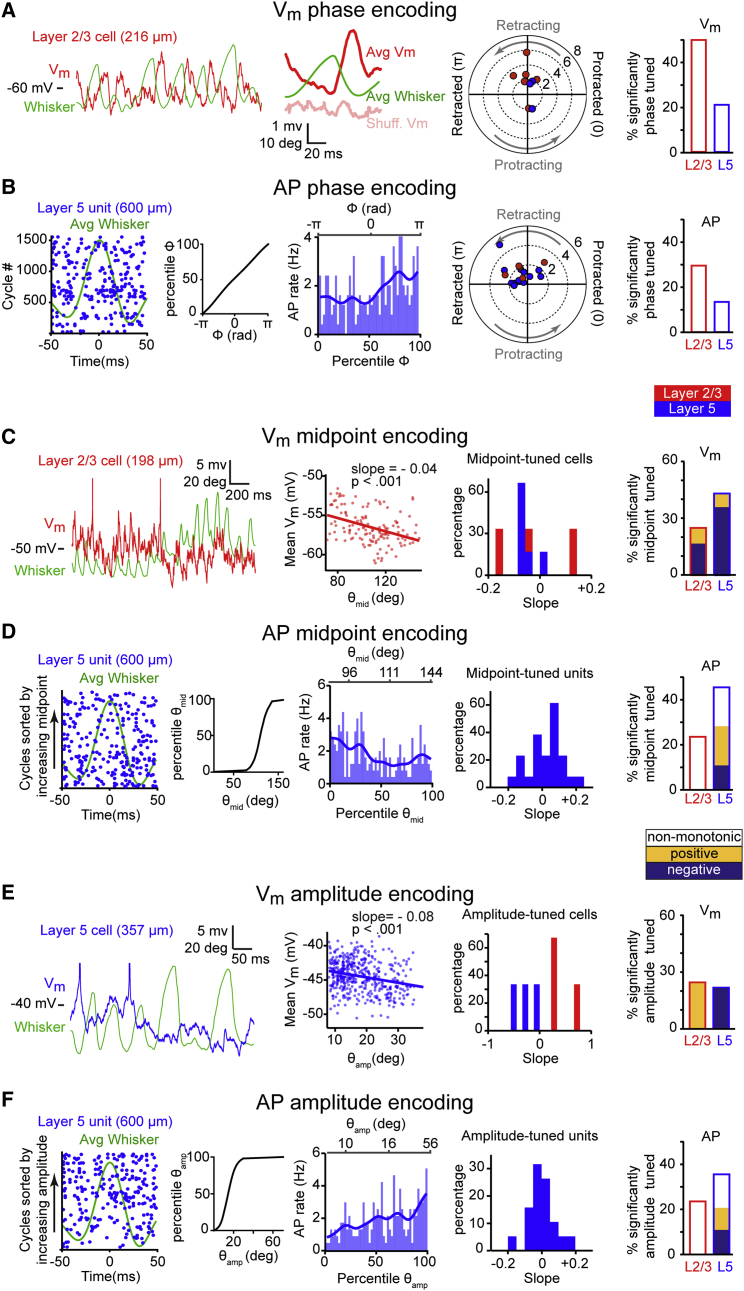
Fast and Slow Whisking Variables Are Encoded in V_m_ and AP Firing of wM1 Neurons (A) Example V_m_ trace (red) from an L2/3 wM1 neuron during whisking (green) (far left). Note the V_m_ modulation coupled to phase of whisk cycle. This V_m_ modulation is also evident in the protraction-triggered average (middle left). Polar plot showing magnitude of V_m_ modulation versus the most depolarized phase in the whisk cycle (middle right). Only cells with significant modulation are indicated for L2/3 (red) and L5 (blue). Percentage of cells with significant V_m_ phase modulation (far right). (B) Example protraction-triggered raster plot of an L5 unit in wM1 (far left). Each row represents a whisk cycle. The inset shows the mapping from phase to percentile. Tuning curve for the unit shown in the left panel (middle left). Note the increase in AP rate during retraction. Polar plot showing magnitude of AP rate modulation versus maximal-firing phase in the whisk cycle for units with significant modulation in L2/3 (red) and L5 (blue) (middle right). Percentage of units with significant AP rate phase modulation (far right). (C) Example V_m_ trace (red) from an L2/3 wM1 neuron during whisking (far left). Note V_m_ hyperpolarization when whisking shifts to a more protracted position. Scatterplot showing mean V_m_ versus whisking midpoint (middle left). Histogram of slopes for cells with significant V_m_ midpoint tuning in L2/3 (red) and L5 (blue) (middle right). Percentage of cells with significant V_m_ midpoint tuning (far right). (D) Example protraction-triggered raster plot of an L5 unit in wM1 sorted by increasing values of midpoint (far left). The inset shows the mapping from midpoint to percentiles. Tuning curve for the unit shown in the left panel (middle left). Note the higher AP rate for smaller whisk midpoints. Histogram of the distribution of slopes for units with significant monotonic AP midpoint tuning (middle right). Only L5 units showed monotonic midpoint tuning. Percentage of units with significant AP rate midpoint tuning (far right). (E) Example V_m_ trace (blue) from an L5 wM1 neuron during whisking (far left). Note V_m_ hyperpolarization when whisking amplitude increases. Scatterplot showing mean V_m_ versus amplitude of whisking for the example cell (middle left). Histogram of distribution of slopes for cells with significant V_m_ amplitude tuning in L2/3 (red) and L5 (blue) (middle right). Percentage of cells with significant V_m_ amplitude tuning (far right). (F) Example protraction-triggered raster plot of an L5 unit in wM1 sorted by increasing values of amplitude (far left). The inset shows the mapping from amplitude to percentiles. Tuning curve for the example unit (middle left). Note the higher AP rate for larger whisk amplitudes. Histogram of the distribution of slopes for units with significant monotonic AP amplitude tuning (middle right). Only L5 units showed monotonic amplitude tuning. Percentage of units with significant AP rate amplitude tuning (far right). See also Figure S3 and Table S3.

We next investigated whether the V_m_ and AP rates of wM1 neurons encode whisker motion during stable bouts of rhythmic whisking. Previous work in rats showed that AP firing in motor cortex encodes a fast whisking variable (phase) and two slow whisking variables (midpoint and amplitude) (Hill et al., 2011). Using a similar strategy, we used the Hilbert transform to decompose whisking epochs into three variables, instantaneous phase (ϕ), midpoint (ϴ_mid_), and amplitude (ϴ_amp_) (Figure S3), and correlated each of these with the V_m_ and AP rates of individual cells and units, respectively, in L2/3 and L5 (Figure 3; Table S3).

Whisking-phase-locked V_m_ fluctuations were prominent in some wM1 neurons (Figure 3A). We found that the V_m_ of a larger fraction of L2/3 neurons (50%, 6/12 cells) was significantly modulated by whisking phase compared to L5 (21.4%, 3/14 cells). Similarly, the fraction of units whose AP firing rate was significantly modulated by whisking phase was larger in L2/3 (29.4%, 5/17 units) compared to L5 (13.3%, 12/90 units) (Figure 3B).

Next, we correlated the V_m_ and AP rates of wM1 cells with the whisking midpoint (ϴ_mid_). Across the population, we observed V_m_-midpoint correlations with both positive and negative slopes (Figure 3C). The fraction of cells whose V_m_ was significantly midpoint modulated was larger in L5 (42.9%, 6/14 cells) compared to L2/3 (25%, 3/12 cells). Similarly, the fraction of units whose AP firing rates were significantly modulated by whisking midpoint was larger in L5 (45.6%, 41/90 units) compared to L2/3 (23.5%, 4/17 units) (Figure 3D).

Finally, we correlated the V_m_ and AP rates of wM1 cells with the whisking amplitude (ϴ_amp_). Across the population, we observed V_m_-amplitude correlations with both positive and negative slopes (Figure 3E). The fraction of cells whose V_m_ was significantly modulated by whisking amplitude was similar between L2/3 (25%, 3/12 units) and L5 (21.4%, 3/14 units). AP firing rate was also significantly modulated by whisking amplitude in L2/3 (23.5%, 4/17 units) and L5 (35.6%, 32/90 units) (Figure 3F).

Thus, all three whisking variables were encoded in V_m_ and AP firing rates of wM1 neurons, consistent with and extending previous results from rat motor cortex (Hill et al., 2011). Interestingly, optogenetic inactivation of wM1 during ongoing whisking rapidly and significantly reduced the amplitude of whisking, suggesting that neuronal activity in wM1 contributes to driving ongoing whisking (Figure S3).

### Optogenetic Inhibition of wS1 Reduces Whisking and Inhibits wM1

Our recordings (Figures 2 and 3) and optogenetic manipulations (Figure 1) demonstrate that the activity of neurons in wM1 correlates and contributes to driving whisker movements. Synaptic input controls the activity of wM1 neurons and, by definition, wM1 receives dense, long-range axonal input from wS1 (Figure 1). Thus, in order to further our understanding of how activity in wM1 is driven, we carried out optogenetic manipulations of wS1 while filming whisker movements and recording neuronal activity in wM1.

**Figure 4:**
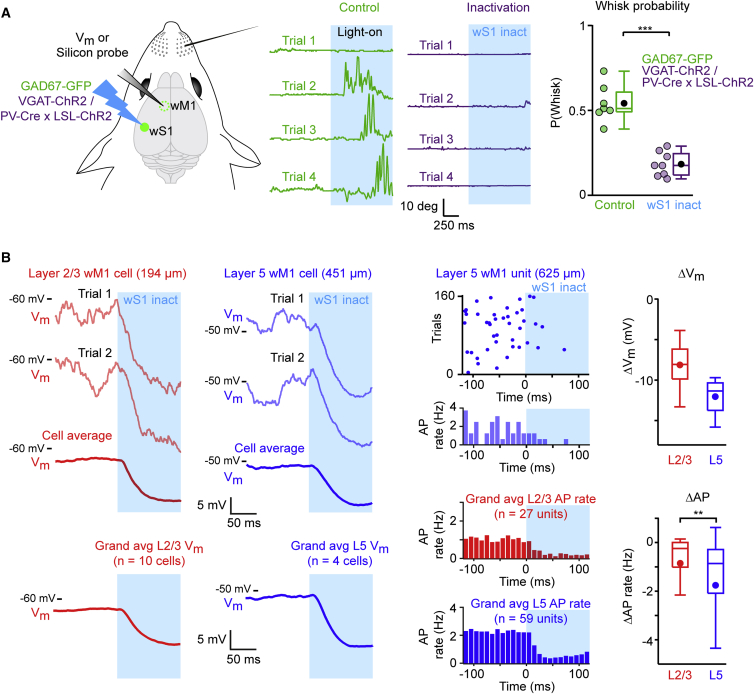
wS1 Inactivation Decreases the Probability of Initiating Whisking, Hyperpolarizes V_m_ in wM1, and Reduces AP Rates in wM1 (A) V_m_ and silicon probe recordings were carried out in wM1 while wS1 was inactivated (left). Four example whisker traces during wS1 inactivation in a VGAT-ChR2 mouse (purple) and control light application in a GAD67-GFP mouse (green) (middle). Only trials without prestimulus whisking were included in the analysis. Quantified across animals, the probability to initiate whisking, P(Whisk), was significantly smaller upon wS1 inactivation compared to control light application (right). Each colored circle corresponds to data from one mouse. Black filled circles show mean. Boxplots indicate median and interquartile range. (B) Example V_m_ traces from L2/3 (red) and L5 (blue) wM1 neurons (upper left). Note rapid hyperpolarization upon wS1 inactivation. Light traces indicate individual trials and dark traces indicate the average. The grand average V_m_ (lower left). Example raster plot and PSTH for an L5 wM1 unit (upper middle). Note drop in AP rates when wS1 is inactivated. The grand average PSTHs (lower middle). wS1 inactivation led to robust hyperpolarization and decreased AP firing in wM1 (right). Filled circles show mean. Boxplots indicate median and interquartile range. See also Figure S4 and Table S4.

We first investigated whether inactivating wS1 resulted in any change in spontaneous whisking (Figure 4A). To this end, we made use of VGAT-ChR2 mice and PV-Cre × LSL-ChR2 mice, where stimulation of ChR2-expressing GABAergic neurons suppresses activity of nearby pyramidal cells (Guo et al., 2014). We specifically analyzed trials in which the mouse was not whisking in the prestimulus baseline period and quantified whisking in the 1 s period during blue light application. Optogenetic inactivation of wS1 led to a significant reduction in the probability of initiating whisking compared to the same light stimulus applied to GAD67-GFP mice (wS1 inactivation median whisk probability during blue light = 0.17, n = 8 mice; GAD67-GFP median whisk probability during blue light = 0.51, n = 7 mice; Wilcoxon-Mann-Whitney test, p = 3 × 10^−4^) (Figure 4A; Table S4).

While inactivating wS1, we measured V_m_ and AP rates of individual wM1 neurons (Figure 4B; Table S4). Opto-inactivation of wS1 led to a rapid (median latency = 10.3 ms, n = 14 cells) and pronounced hyperpolarization of V_m_ in wM1 (median ΔV_m_ = −9.1 mV, n = 14 cells; Wilcoxon signed-rank test, p = 1.2 × 10^−4^), which was prominent in both L2/3 (median ΔV_m_ = −8 mV, n = 10 cells) and L5 (median ΔV_m_ = −11.3 mV, n = 4 cells) (Figure 4B). This V_m_ hyperpolarization was accompanied by a rapid (median latency to drop in AP rate = 10 ms, n = 86 wM1 units; Wilcoxon signed-rank test, p = 2 × 10^−9^) and strong decrease in AP firing rate in wM1 (median ΔAP = −0.67 Hz, n = 86 wM1 units; Wilcoxon signed-rank test, p = 5 × 10^−13^), and this decrease was significantly larger in L5 (median ΔAP; L2/3 = −0.24 Hz, n = 27 units; L5 = −0.86 Hz, n = 59 units; Wilcoxon-Mann-Whitney test, p = 0.01) (Figure 4B). Fast-spiking units in wM1 also reduced AP firing rates, thus ruling out the possibility of local inhibition causing the suppression of AP rates in wM1 excitatory units (median ΔAP = −3.77 Hz, n = 14 wM1 fast-spiking units; Wilcoxon signed-rank test, p = 1 × 10^−4^) (Figure S4).

Thus, activity in wS1 appears to contribute to an important ongoing excitatory drive to wM1 neurons, thereby keeping the V_m_ of individual wM1 cells depolarized with elevated AP firing rates. Inactivation of wS1 leads to a rapid hyperpolarization of V_m_ and reduction in AP firing rate in wM1 that likely contribute to the reduced probability of initiating whisking.

### wS1 Stimulation Evokes Delayed Whisking following a Complex Triphasic Response in wM1

**Figure 5:**
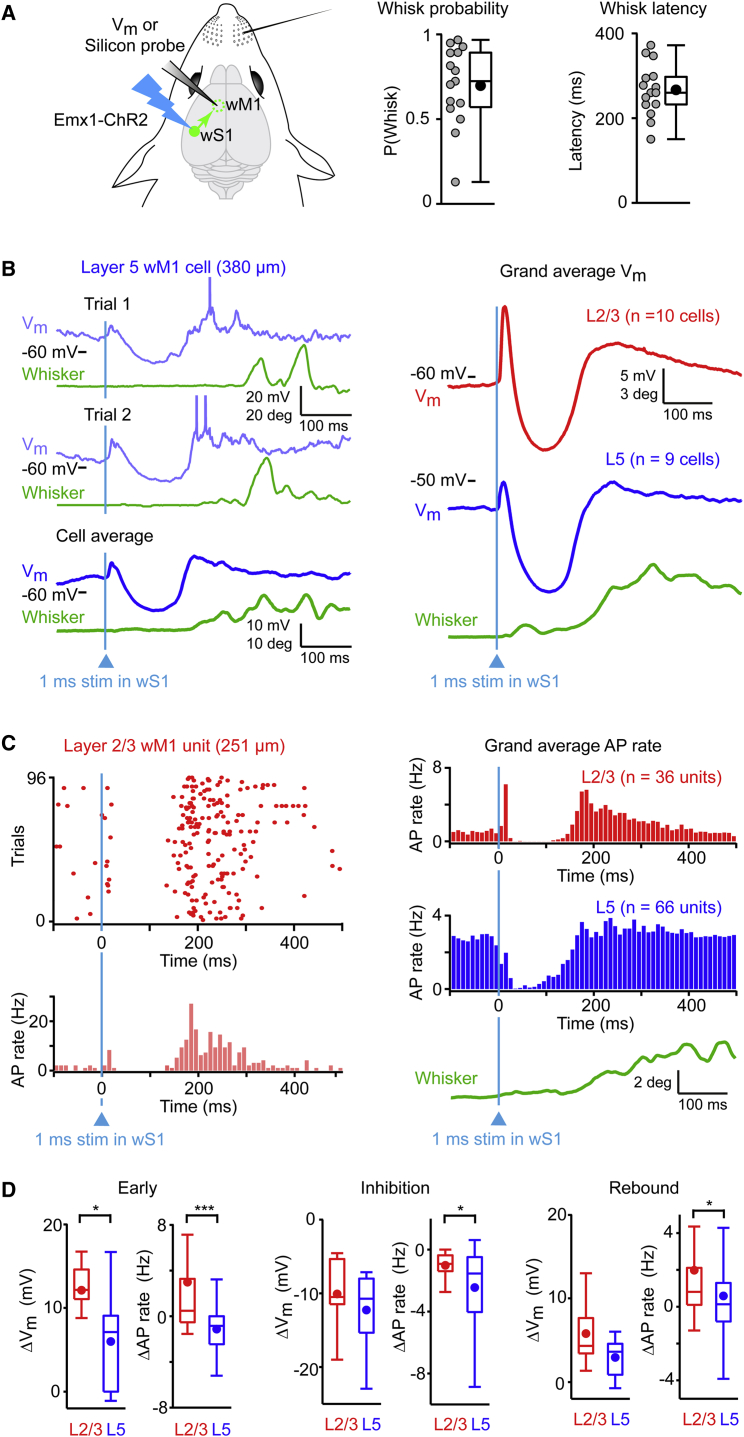
wS1 Activation Generates a Triphasic Response in wM1 Leading to Initiation of Whisking (A) V_m_ and silicon probe recordings were carried out in wM1 while wS1 was optogenetically excited with a 1 ms blue light pulse (left). wS1 stimulation led to whisker movement initiation with long latencies (right). (B) Example V_m_ traces (blue) from an L5 wM1 neuron upon wS1 activation (left). Note the triphasic V_m_ response with whisker movement initiation (green) following rebound depolarization. Lighter traces indicate individual trials while dark trace indicates average across trials for that cell. Grand average V_m_ response (right). (C) Example raster plot and PSTH (red) for an L2/3 wM1 unit upon wS1 stimulation (left). Grand average PSTHs (right). Note triphasic AP response and initiation of whisking (green) following third phase. (D) Quantification of the change in V_m_ and AP rate with respect to baseline during Early (left), Inhibition (middle), and Rebound (right) phases. Filled circles show mean. Boxplots indicate median and interquartile range. See also Figure S5 and Table S5.

We next investigated the effect of stimulating wS1 upon whisking and neuronal activity in wM1. We injected Cre-dependent AAV to express ChR2 in excitatory neurons in wS1 of Emx1-Cre mice. A brief (1 ms) blue light flash applied to wS1 evoked whisking with high probability (median whisk probability = 0.72, n = 15 mice), but with a relatively long latency (median latency = 260 ms, n = 15 mice) (Figure 5A; Table S5). At lower stimulus strength, the probability of evoking whisking decreased and latency increased (Figure S5), whereas at higher stimulation strengths a fast retraction of the contralateral whisker precedes the long-latency whisking (Matyas et al., 2010, Sreenivasan et al., 2015). The long latency for evoking whisker movement is surprising, given that wS1 strongly innervates wM1, and that wM1 drives short-latency whisker movement. We therefore carried out whole-cell V_m_ recordings (Figure 5B) and silicon probe AP measurements (Figure 5C) in wM1 to investigate the temporal dynamics of the evoked response.

Shortly after optogenetic stimulation, neurons in wM1 depolarized, presumably driven by the direct monosynaptic excitatory input from wS1. This “Early” depolarization was significantly larger in L2/3 compared to L5 cells (median ΔV_m_; L2/3 = 12.1 mV, n = 10 cells; L5 = 7.1 mV, n = 9 cells; Wilcoxon-Mann-Whitney test, p = 0.017) (Figures 5B and 5D; Table S5). Early AP firing rates (quantified from 0 to 20 ms after wS1 stimulation) also increased significantly in L2/3, but not in L5 (median ΔAP; L2/3 = 0.48 Hz, n = 36 units; L5 = −0.84 Hz, n = 66 units; Wilcoxon-Mann-Whitney test, p = 1.2 × 10^−5^) (Figures 5C and 5D; Table S5). The overall paucity of evoked APs is likely due to the rapid recruitment of local inhibition in wM1, as suggested by a “reversal potential,” which was hyperpolarized relative to AP threshold for most neurons (median V_rev_ = −43.1 mV; median AP threshold = −34.4 mV; n = 18 cells; Wilcoxon signed-rank test, p = 0.015), thus preventing AP initiation (Crochet et al., 2011, Mateo et al., 2011) (Figure S5; Table S5).

This Early phase was rapidly curtailed by a phase of “Inhibition,” with hyperpolarization relative to prestimulus baseline in both L2/3 and L5 cells (median ΔV_m_; L2/3 = −10.5 mV, n = 10 cells; L5 = −10.7 mV, n = 9 cells; Wilcoxon-Mann-Whitney test, p = 0.49) (Figures 5B and 5D; Table S5). While this hyperpolarization did not differ significantly comparing the two layers, the decrease in AP firing rate (quantified from 20 to 120 ms after wS1 stimulation) was significantly stronger in L5 compared to L2/3 (median ΔAP; L2/3 = −0.92 Hz, n = 36 units; L5 = −1.54 Hz, n = 66 units; Wilcoxon-Mann-Whitney test, p = 0.01) (Figures 5C and 5D; Table S5).

Following the Inhibition phase, the V_m_ depolarized again, leading to a late “Rebound” excitation (quantified from 200 to 300 ms following wS1 stimulation relative to prestimulus baseline). This Rebound depolarization was prominent in both L2/3 and L5 neurons (median ΔV_m_; L2/3 = 4.3 mV, n = 10 cells; L5 = 3.6 mV, n = 9 cells; Wilcoxon-Mann-Whitney test, p = 0.11) (Figures 5B and 5D; Table S5). The increase in AP firing rates during the Rebound phase was significantly larger in L2/3 compared to L5 (median ΔAP; L2/3 = 0.80 Hz, n = 36 units; L5 = 0.13 Hz, n = 66 units, Wilcoxon-Mann-Whitney test, p = 0.01) (Figures 5C and 5D; Table S5).

### wS1-Evoked Rebound Spiking in wM1 Correlates with Initiation of Whisking

**Figure 6:**
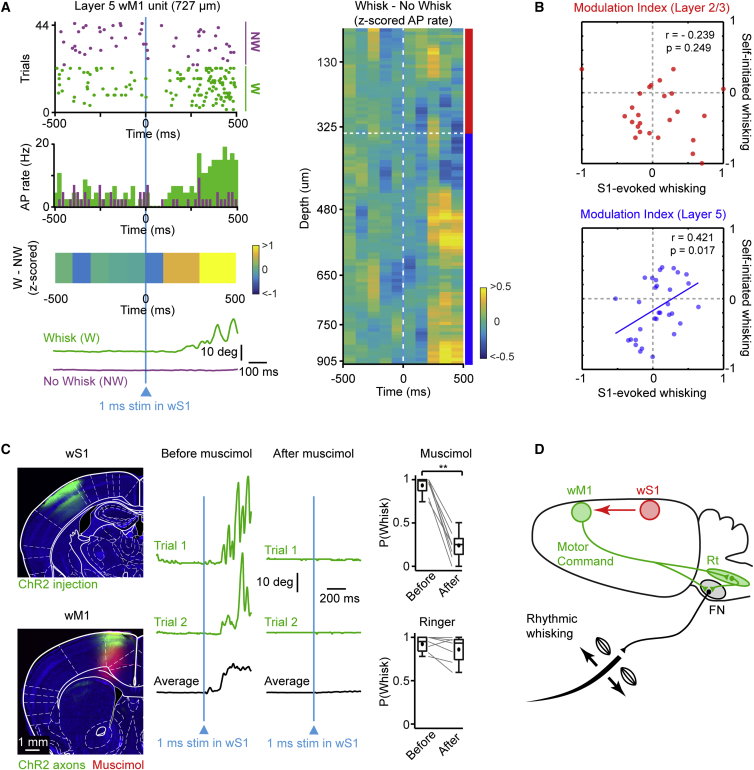
Whisking Evoked by wS1 Stimulation Depends upon wM1 and Correlates with Activity in Specific Subsets of L5 Neurons (A) Example raster plot and PSTH for an L5 wM1 unit upon 1 ms optogenetic stimulation of wS1. The trials are grouped depending on whether the stimulus initiated whisking (Whisk, green) or not (No Whisk, magenta) (upper left). Note increase in AP rate during Rebound on Whisk trials, but not on No Whisk trials. The color-coded normalized z-scored AP difference between Whisk and No Whisk trials for the example unit, together with average whisker traces (lower left). *Z* score activity map (Whisk – No Whisk) for all wM1 units (right). Note prominent positive AP rate difference in L5. (B) Scatterplot of AP modulation index during wS1-evoked whisking versus self-initiated whisking; each circle represents a single unit. The modulation indices did not correlate in L2/3 (top) but positively correlated in L5 (bottom), indicating that L5 neurons that are modulated during wS1-evoked whisking also tend to be similarly modulated during self-initiated whisking. (C) ChR2 (green) was expressed in wS1, and muscimol (red) was injected into wM1 (left). Example whisker traces (green) upon wS1 stimulation before and after muscimol inactivation of wM1 (middle). Quantified across animals, muscimol inactivation of wM1 significantly reduced the probability of initiating whisking upon wS1 activation (upper right). Injection of Ringer’s solution in wM1 did not affect initiation of whisking (lower right). Gray lines indicate individual mice and black circles indicate mean. Boxplots indicate median and interquartile range. (D) Schematic drawing of the wS1→wM1 sensorimotor circuit. wM1 initiates rhythmic whisking by issuing a motor command to brainstem circuitry (Rt, reticular formation; FN, facial nucleus). wS1 in turn provides tonic excitatory drive to wM1 and can trigger wM1 activation, thereby initiating rhythmic whisking. See also Figure S6 and Table S6.

Exploratory whisking was initiated after the beginning of the Rebound phase (Figure S6), and we therefore hypothesized that the Rebound firing contributed causally to whisking initiation. Interestingly, whisker movements were not initiated on every wS1 stimulation trial, even when the same stimulus was repeatedly applied. We therefore compared trials in which mice initiated whisking following the stimulus (“Whisk” trials) with trials in which they did not initiate whisking following the same stimulus (“No Whisk” trials). Some neurons showed a striking difference in AP firing during the Rebound period comparing Whisk and No Whisk trials (Figures 6A and S6). Plotting the z-scored difference in AP rates across the entire population of recorded units, we found that Rebound firing in L5 neurons was larger on Whisk trials compared to No Whisk trials, whereas this was less evident in L2/3 (Figures 6A and S6). However, the increase in firing was rather heterogeneous and we therefore investigated the relationship between rebound activity and AP firing rate modulation by spontaneous whisking in individual neurons. We correlated the AP modulation index during wS1-evoked whisking with the modulation index during spontaneous whisking for L2/3 and L5 units (Figure 6B). The modulation indices were not correlated for L2/3 (r = −0.24; p = 0.25, permutation test) but were significantly correlated for L5 units (r = 0.42; p = 0.02, permutation test) (Figure 6B; Table S6). These results indicate that L5, but not L2/3, units are modulated similarly during wS1-evoked and self-initiated whisking. AP firing during the late Rebound phase in specific whisking-related populations of L5 neurons might thus serve as a motor command in wM1 to initiate whisking.

### wS1-Evoked Whisking Requires wM1

Finally, we directly tested the need for wM1 in initiating whisking upon wS1 stimulation by pharmacological inactivation of wM1. To this end, we stimulated wS1 while recording whisker movements, before and after injection of muscimol, a GABA_A_-receptor agonist, in wM1. Muscimol inactivation of wM1 led to a dramatic drop in the probability of initiating whisking upon wS1 stimulation (median whisk probability, before muscimol = 0.98 versus after muscimol = 0.25, n = 8 mice; Wilcoxon signed-rank test, p = 0.008) (Figure 6C; Table S6). Injection of Ringer’s solution in wM1 did not affect the probability of initiating whisking (median whisk probability, before Ringer = 0.95 versus after Ringer = 0.93, n = 7 mice; Wilcoxon signed-rank test, p = 0.22) (Figure 6C; Table S6). Our results thus suggest that activity in wM1 is required to initiate exploratory whisking following wS1 stimulation (Figure 6D).

## Discussion

In this study, we investigated an anatomically defined frontal region, wM1, which receives strong innervation from wS1, and, using optogenetics, we demonstrated a causal role for this region in initiating whisker movements (Figure 1). Whole-cell and silicon probe recordings revealed that excitation of L5 neurons in wM1 preceded the initiation of spontaneous whisking (Figure 2). Immediately after the onset of whisking, L2/3 neurons in wM1 were inhibited, and the activity of a large fraction of L5 neurons was reorganized (Figure 2). During bouts of self-generated whisking, wM1 neurons encoded three key whisking variables (Figure 3). Optogenetic inactivation revealed that ongoing activity in wS1 contributed strongly to the excitation of wM1 neurons and the initiation of whisking (Figure 4). Conversely, optogenetic stimulation of wS1 evoked a triphasic response in wM1, following which the mouse began to whisk, if whisking-related neurons in wM1 were appropriately activated (Figures 5 and 6). Together, our results begin to shed light on how whisker movements might be initiated and controlled by motor cortex, highlighting an important role for input from sensory cortex.

### Motor Commands for Initiation of Whisking in wM1

Changes in neuronal activity preceding movement initiation could serve as motor commands, and such changes, preceding volitional hand movements, have been demonstrated in motor cortex of primates (Georgopoulos et al., 1986) and humans (Goldring and Ratcheson, 1972). In the mouse, whisker movements can be initiated at short latencies (∼25 ms) following stimulation of wM1 (Figure 1), and one might therefore expect motor commands for initiation of whisking immediately before whisking onset. We found significant depolarization and increases in AP firing across the population of recorded L5 wM1 neurons in the 100 ms period before initiation of whisking (Figure 2). Almost a fifth of all L5 wM1 neurons significantly increased firing rate in this pre-movement period, whereas only 4% decreased firing rate in this period. L5 neurons of wM1 prominently innervate brainstem reticular formation, which harbors whisker premotor neurons (Takatoh et al., 2013, Sreenivasan et al., 2015), and also directly innervate the whisker motor neurons in the facial nucleus (Grinevich et al., 2005, Sreenivasan et al., 2015) (Figure 6D). The increased firing of L5 wM1 neurons immediately before initiation of whisking may therefore serve as a motor command. In future studies, it will be important to distinguish different types of L5 neurons, to be able to specifically address whether pyramidal tract neurons are excited before whisking onset.

The source of the depolarization and excitation of the L5 wM1 neurons preceding whisker movement is currently unknown. L5 wM1 neurons receive important input from L2/3 wM1 neurons (Hooks et al., 2011). Although there was no significant depolarization or increase in AP firing across the population of L2/3 wM1 neurons, individual L2/3 wM1 neurons were significantly excited in the pre-movement period; 10.8% of L2/3 wM1 neurons showed a significant increase in firing rate. The depolarization of L5 wM1 neurons in the pre-movement period may thus in part be driven by increased firing of a specific group of L2/3 wM1 neurons. Apart from local trans-laminar input, L5 wM1 neurons also receive significant excitatory synaptic input from secondary motor cortex and anterior motor thalamus (Mao et al., 2011, Hooks et al., 2013), all of which could contribute importantly to the generation of the whisking initiation motor command. Neuromodulatory input might also play a role; for example, it is possible that wM1 receives a whisking-related cholinergic input, similar to wS1 (Eggermann et al., 2014), which might also have an important effect. Future experiments must therefore investigate the roles of the diverse synaptic inputs to L5 wM1 neurons in driving pre-movement depolarization and increased firing.

### Coding of Whisker Movement in wM1

The overall increase in AP rates for L5 wM1 neurons at whisking onset was transient: the average firing rate returned to baseline levels ∼200 ms after the initiation of whisking. Although there was no sustained increase in AP rates during whisking, there is nonetheless a very important reorganization of which neurons are active during whisking compared to baseline non-whisking periods (Figure 2). More than half of the L2/3 neurons in wM1 are significantly inhibited during whisking, and approximately half of the L5 neurons have a significantly increased or decreased firing rate. The pattern of network activity in wM1 is therefore very different comparing whisking and non-whisking periods. A large fraction of the neurons that are active during whisking encode different aspects of the ongoing whisker movements. Consistent with previous findings in rat motor cortex (Hill et al., 2011), we found that neuronal activity in mouse wM1 encodes detailed information about whisker position on fast as well as slow timescales. These signals might be motor related, contributing to controlling whisker movement; they may result from sensory reafference; or they may be mixed sensory and motor signals.

Interestingly, a larger proportion of L2/3 wM1 neurons were strongly modulated by the whisking phase compared to L5 wM1 neurons. L2/3 wM1 is thought to be the most important recipient layer for sensory information from wS1 (Mao et al., 2011). AP firing (Curtis and Kleinfeld, 2009) and V_m_ fluctuations in wS1 (Crochet and Petersen, 2006) correlate with rhythmic whisker movements. In wS1, these phase-locked oscillations are abolished upon transecting the infraorbital nerve, indicating that the source of these fluctuations might be re-afferent signals from the periphery (Poulet and Petersen, 2008), presumably relayed via the primary somatosensory ventral posterior medial (VPM) thalamic nucleus (Moore et al., 2015, Urbain et al., 2015). Interestingly, phase-locked fluctuations in wM1-projecting neurons of wS1 are significantly larger than those in wS2-projecting neurons of wS1 (Yamashita et al., 2013). The fast phase-locked V_m_ fluctuations and AP modulation in wM1 are thus likely, at least in part, to be due to peripheral re-afference relayed to wM1 via wS1. In the future, experiments transecting the sensory nerve at the periphery will be important to determine the relative contributions of sensory re-afference compared to internal motor commands in wM1 (Hill et al., 2011).

### Sensory Control of wM1

Optogenetic inactivation of wS1 had a striking impact upon wM1. With a short latency of ∼10 ms, neurons in wM1 began to hyperpolarize and reduce AP firing rates. Putative inhibitory neurons in wM1 also reduced firing rates, indicating that the hyperpolarization and suppression of activity were likely due to a loss of excitatory input to wM1. Excitatory pyramidal neurons in wS1 strongly innervate L2/3 of wM1 (Mao et al., 2011), and therefore inactivation of wS1 should remove ongoing excitatory input to L2/3 wM1 neurons, consistent with our measurements. Reduced firing in L2/3 of wM1 will, in turn, reduce excitatory input to L5 wM1, since this is the major excitatory synaptic pathway within the wM1 microcircuit (Hooks et al., 2011). Hyperpolarization and reduced firing of L5 wM1 neurons might therefore be a secondary knock-on effect induced by wS1 inactivation. The reduced firing of L5 wM1 neurons during wS1 inactivation is likely to contribute to the reduced probability of initiating whisking.

Many other polysynaptic pathways originating from wS1 could contribute to the suppression of wM1 following wS1 inactivation. For example, the secondary somatosensory thalamic nucleus, POm, will also receive less excitation from wS1 when it is inactivated (Mease et al., 2016), and POm also projects to wM1 (Hooks et al., 2013). It is therefore possible that the massive impact of wS1 inactivation upon wM1 is mediated by a self-amplifying inhibition. Here, we provide clear evidence that cortical regions downstream of an inactivated area can be strongly affected, and our data therefore relate to “off-target” effects of optogenetic inactivation (Otchy et al., 2015), suggesting that these experiments require careful interpretation.

Optogenetic stimulation of wS1 also had a profound impact upon wM1. Stimulation of wS1 evoked a short-latency depolarizing response accompanied by increased AP firing in some L2/3 neurons of wM1, consistent with the synaptic connectivity measured in vitro (Mao et al., 2011). However, neurons in L5 of wM1 were inhibited, showing reduced firing rates. It is likely that inhibitory GABAergic neurons in wM1 are strongly excited by the optogenetic stimulation of wS1, similar to the effect on local wS1 microcircuits, in which inhibition is the dominant postsynaptic response to stimulation of excitatory neurons (Mateo et al., 2011). The recruitment of local GABAergic neurons in wM1 by the optogenetic stimulation of wS1 likely explains the hyperpolarized reversal potentials of the wS1-evoked response in wM1 and the small number of evoked APs in wM1. Given that L5 wM1 neurons were rapidly inhibited by the optogenetic stimulation of wS1, it is perhaps not surprising that there is little immediate behavioral effect in terms of whisking. Activity in wM1 returns after a period of inhibition, and after this rebound excitation period, the mouse is likely to initiate whisking. The activity of wM1 is essential for this, since the mouse will only rarely whisk in response to wS1 stimulation if wM1 is inactivated. Whether the mouse initiates whisking appears to depend upon which neurons in wM1 become activated during the rebound period. During volitional self-generated whisking, some L5 wM1 neurons are excited and others are inhibited. If these same neurons are modulated in the same way after wS1 stimulation, then the mouse begins to whisk (Figure 6). In the future, it will be important to investigate the projection targets of these “whisking” neurons in wM1, what mechanisms drive the late rebound activity, and whether they form specific competing ensembles (Zagha et al., 2015).

Optogenetic stimulation of wS1 is obviously a highly artificial stimulus, but in some respects it closely mimics the response to passive and active whisker deflection, which both evoke brief, transient AP firing in wS1 neurons projecting to wM1 (Yamashita et al., 2013, Yamashita and Petersen, 2016). Passive deflection of a whisker can evoke whisking (Ferezou et al., 2007, Yamashita et al., 2013), similar to the effects observed here with optogenetic stimulation of wS1. Our results showing that optogenetic stimulation of wS1 evokes whisking are therefore likely to relate to the mechanisms by which a peripheral sensory stimulus evokes a volitional motor reaction.

### Future Perspectives

Our data are not easy to reconcile with the recently published findings of Ebbesen et al. (2016), who suggest that “vibrissa motor cortex activity suppresses contralateral whisking behavior.” There are important methodological differences between the studies, including the species investigated, the cortical region being recorded, and the methods for cortical stimulation and inactivation. Whereas our study focuses on signals in wM1 underlying initiation of whisking in head-restrained mice, the study of Ebbesen et al. (2016) focused on control of ongoing whisker movements in rats during complex behavior. It is possible that different brain regions contribute differentially to the control of whisker movements depending upon behavioral context. Premotor neurons for whisker motor control are widely distributed, and it is likely that there are many brain regions involved in controlling whisker movement. Here, we have focused on one region (wM1) that contributes importantly to controlling whisker movements under our experimental conditions, but other brain regions might dominate during different behaviors, for example, during running and locomotion. Further research is necessary before we understand the organization and function of wM1 and other brain regions involved in whisker motor control. Defining the specific activities of different types of neurons during different behaviors will help toward mechanistic understanding.

It is also interesting to note that the same apparent cortical region appears to play a role in orienting (Erlich et al., 2011), licking in a learned whisker-dependent task (Huber et al., 2012), and rotor-rod performance (Cao et al., 2015). It is therefore possible that whisker motor control is only a part of the overall function of wM1. Indeed, there are important open questions about the organization of frontal cortical regions, which, although they are often thought to contain well-ordered motor maps (Fritsch and Hitzig, 1870, Penfield and Boldrey, 1937, Brecht et al., 2004), could in fact be organized according to different principles into behaviorally related modules, such as the “action zones” proposed for macaque motor cortex (Graziano et al., 2002).

## Experimental Procedures

### Animal Preparation and Surgery

All experiments were carried out in accordance with protocols approved by the Swiss Federal Veterinary Office. Adult 6- to 9-week-old male and female mice were implanted with a light-weight metal head post under isoflurane anesthesia. Following recovery, they were habituated to head restraint.

### Optogenetics

Optogenetic activation experiments were carried out by expressing ChR2 using a Cre-dependent virus injected into Emx1-Cre mice (RRID: IMSR_JAX:005628). Optogenetic inactivation experiments were carried out in VGAT-ChR2 mice (RRID: IMSR_JAX:014548) or PV-Cre × LSL-ChR2 mice (PV-Cre, RRID: IMSR_JAX:008069; LSL-ChR2, RRID: IMSR_JAX:012569). The stimulus was delivered through a 400 μm fiber-optic cable coupled to a 470 nm high-power LED.

### Electrophysiology

In vivo whole-cell recordings were targeted to wM1 in awake head-restrained mice. The pipette internal solution contained 135 mM potassium gluconate, 4 mM KCl, 10 mM sodium phosphocreatinine, 4 mM MgATP, 0.3 mM Na_3_GTP, 10 mM HEPES (pH 7.3), and 2–4 mg/mL biocytin. The membrane potential was recorded without current injection. Liquid junction potential was not corrected.

Extracellular spikes were recorded using a silicon probe with 32 recording sites. The probe was coated with DiI for post hoc recovery of recording location, and then lowered gradually into wM1. Spiking activity was detected and sorted into different clusters using KlustaSuite (Rossant et al., 2016).

### Whisker Filming

Whisker movements were filmed at 500 Hz. All whiskers were trimmed except the C2 whiskers on either side. Whisker angle was quantified using custom routines.

### Statistics

All group data are presented as boxplots. On each box, the central mark indicates median, and the edges of the box indicate 25th and 75th percentiles. The whiskers extend to the most extreme data points, excluding outliers. The mean is also indicated. Statistical testing was carried out in MATLAB. All group comparisons were performed using Wilcoxon signed-rank or Wilcoxon-Mann-Whitney tests. Analysis of individual neurons was performed using non-parametric permutation tests.

## Author Contributions

V.S., V.E., S.C., and C.C.H.P. designed the project and wrote the manuscript. V.S. and V.E. performed electrophysiological recordings and optogenetic experiments, and analyzed data. T.K. contributed to high-speed whisker filming. K.G. contributed to histology.

## Supplemental Information

Document S1. Supplemental Experimental Procedures, Figures S1–S6, and Tables S1–S6

Document S2. Article plus Supplemental Information

## References

[bib1] Aronoff R., Matyas F., Mateo C., Ciron C., Schneider B., Petersen C.C.H. (2010). Long-range connectivity of mouse primary somatosensory barrel cortex. Eur. J. Neurosci..

[bib2] Brecht M., Krauss A., Muhammad S., Sinai-Esfahani L., Bellanca S., Margrie T.W. (2004). Organization of rat vibrissa motor cortex and adjacent areas according to cytoarchitectonics, microstimulation, and intracellular stimulation of identified cells. J. Comp. Neurol..

[bib3] Cao V.Y., Ye Y., Mastwal S., Ren M., Coon M., Liu Q., Costa R.M., Wang K.H. (2015). Motor learning consolidates Arc-expressing neuronal ensembles in secondary motor cortex. Neuron.

[bib4] Crochet S., Petersen C.C.H. (2006). Correlating whisker behavior with membrane potential in barrel cortex of awake mice. Nat. Neurosci..

[bib5] Crochet S., Poulet J.F.A., Kremer Y., Petersen C.C.H. (2011). Synaptic mechanisms underlying sparse coding of active touch. Neuron.

[bib6] Curtis J.C., Kleinfeld D. (2009). Phase-to-rate transformations encode touch in cortical neurons of a scanning sensorimotor system. Nat. Neurosci..

[bib7] Deschênes M., Takatoh J., Kurnikova A., Moore J.D., Demers M., Elbaz M., Furuta T., Wang F., Kleinfeld D. (2016). Inhibition, not excitation, drives rhythmic whisking. Neuron.

[bib8] Diamond M.E., von Heimendahl M., Knutsen P.M., Kleinfeld D., Ahissar E. (2008). ‘Where’ and ‘what’ in the whisker sensorimotor system. Nat. Rev. Neurosci..

[bib9] Ebbesen C.L., Doron G., Lenschow C., Brecht M. (2016). Vibrissa motor cortex activity suppresses contralateral whisking behavior. Nat. Neurosci..

[bib10] Eggermann E., Kremer Y., Crochet S., Petersen C.C.H. (2014). Cholinergic signals in mouse barrel cortex during active whisker sensing. Cell Rep..

[bib11] Erlich J.C., Bialek M., Brody C.D. (2011). A cortical substrate for memory-guided orienting in the rat. Neuron.

[bib12] Evarts E.V. (1968). Relation of pyramidal tract activity to force exerted during voluntary movement. J. Neurophysiol..

[bib13] Ferezou I., Haiss F., Gentet L.J., Aronoff R., Weber B., Petersen C.C.H. (2007). Spatiotemporal dynamics of cortical sensorimotor integration in behaving mice. Neuron.

[bib14] Ferrier D. (1874). Experiments on the brain of monkeys–No. 1. Proc. R. Soc. Lond..

[bib15] Fetz E.E., Finocchio D.V., Baker M.A., Soso M.J. (1980). Sensory and motor responses of precentral cortex cells during comparable passive and active joint movements. J. Neurophysiol..

[bib16] Friedman W.A., Zeigler H.P., Keller A. (2012). Vibrissae motor cortex unit activity during whisking. J. Neurophysiol..

[bib17] Fritsch G., Hitzig E. (1870). Über die elektrische Erregbarkeit des Grosshirns. Arch. Anat. Physiol. Wissen..

[bib18] Gentet L.J., Avermann M., Matyas F., Staiger J.F., Petersen C.C.H. (2010). Membrane potential dynamics of GABAergic neurons in the barrel cortex of behaving mice. Neuron.

[bib19] Georgopoulos A.P., Schwartz A.B., Kettner R.E. (1986). Neuronal population coding of movement direction. Science.

[bib20] Gerdjikov T.V., Haiss F., Rodriguez-Sierra O.E., Schwarz C. (2013). Rhythmic whisking area (RW) in rat primary motor cortex: an internal monitor of movement-related signals?. J. Neurosci..

[bib21] Goldring S., Ratcheson R. (1972). Human motor cortex: sensory input data from single neuron recordings. Science.

[bib22] Graziano M.S., Taylor C.S., Moore T. (2002). Complex movements evoked by microstimulation of precentral cortex. Neuron.

[bib23] Grinevich V., Brecht M., Osten P. (2005). Monosynaptic pathway from rat vibrissa motor cortex to facial motor neurons revealed by lentivirus-based axonal tracing. J. Neurosci..

[bib24] Guo Z.V., Li N., Huber D., Ophir E., Gutnisky D., Ting J.T., Feng G., Svoboda K. (2014). Flow of cortical activity underlying a tactile decision in mice. Neuron.

[bib25] Hill D.N., Curtis J.C., Moore J.D., Kleinfeld D. (2011). Primary motor cortex reports efferent control of vibrissa motion on multiple timescales. Neuron.

[bib26] Hooks B.M., Hires S.A., Zhang Y.X., Huber D., Petreanu L., Svoboda K., Shepherd G.M. (2011). Laminar analysis of excitatory local circuits in vibrissal motor and sensory cortical areas. PLoS Biol..

[bib27] Hooks B.M., Mao T., Gutnisky D.A., Yamawaki N., Svoboda K., Shepherd G.M. (2013). Organization of cortical and thalamic input to pyramidal neurons in mouse motor cortex. J. Neurosci..

[bib28] Huber D., Gutnisky D.A., Peron S., O’Connor D.H., Wiegert J.S., Tian L., Oertner T.G., Looger L.L., Svoboda K. (2012). Multiple dynamic representations in the motor cortex during sensorimotor learning. Nature.

[bib29] Jones E.G., Coulter J.D., Hendry S.H.C. (1978). Intracortical connectivity of architectonic fields in the somatic sensory, motor and parietal cortex of monkeys. J. Comp. Neurol..

[bib30] Mao T., Kusefoglu D., Hooks B.M., Huber D., Petreanu L., Svoboda K. (2011). Long-range neuronal circuits underlying the interaction between sensory and motor cortex. Neuron.

[bib31] Mateo C., Avermann M., Gentet L.J., Zhang F., Deisseroth K., Petersen C.C.H. (2011). In vivo optogenetic stimulation of neocortical excitatory neurons drives brain-state-dependent inhibition. Curr. Biol..

[bib32] Matyas F., Sreenivasan V., Marbach F., Wacongne C., Barsy B., Mateo C., Aronoff R., Petersen C.C.H. (2010). Motor control by sensory cortex. Science.

[bib33] Mease R.A., Sumser A., Sakmann B., Groh A. (2016). Cortical dependence of whisker responses in posterior medial thalamus in vivo. Cereb. Cortex.

[bib34] Mitchinson B., Martin C.J., Grant R.A., Prescott T.J. (2007). Feedback control in active sensing: rat exploratory whisking is modulated by environmental contact. Proc. Biol. Sci..

[bib35] Moore J.D., Deschênes M., Furuta T., Huber D., Smear M.C., Demers M., Kleinfeld D. (2013). Hierarchy of orofacial rhythms revealed through whisking and breathing. Nature.

[bib36] Moore J.D., Mercer Lindsay N., Deschênes M., Kleinfeld D. (2015). Vibrissa self-motion and touch are reliably encoded along the same somatosensory pathway from brainstem through thalamus. PLoS Biol..

[bib37] Otchy T.M., Wolff S.B.E., Rhee J.Y., Pehlevan C., Kawai R., Kempf A., Gobes S.M.H., Ölveczky B.P. (2015). Acute off-target effects of neural circuit manipulations. Nature.

[bib38] Penfield W., Boldrey E. (1937). Somatic motor and sensory representation in the cerebral cortex of man as studied by electrical stimulation. Brain.

[bib39] Petersen C.C.H. (2007). The functional organization of the barrel cortex. Neuron.

[bib40] Petersen C.C.H. (2014). Cortical control of whisker movement. Annu. Rev. Neurosci..

[bib41] Poulet J.F.A., Petersen C.C.H. (2008). Internal brain state regulates membrane potential synchrony in barrel cortex of behaving mice. Nature.

[bib42] Rossant C., Kadir S.N., Goodman D.F.M., Schulman J., Hunter M.L.D., Saleem A.B., Grosmark A., Belluscio M., Denfield G.H., Ecker A.S. (2016). Spike sorting for large, dense electrode arrays. Nat. Neurosci..

[bib43] Semba K., Komisaruk B.R. (1984). Neural substrates of two different rhythmical vibrissal movements in the rat. Neuroscience.

[bib44] Sreenivasan V., Karmakar K., Rijli F.M., Petersen C.C.H. (2015). Parallel pathways from motor and somatosensory cortex for controlling whisker movements in mice. Eur. J. Neurosci..

[bib45] Takatoh J., Nelson A., Zhou X., Bolton M.M., Ehlers M.D., Arenkiel B.R., Mooney R., Wang F. (2013). New modules are added to vibrissal premotor circuitry with the emergence of exploratory whisking. Neuron.

[bib46] Urbain N., Salin P.A., Libourel P.A., Comte J.C., Gentet L.J., Petersen C.C.H. (2015). Whisking-related changes in neuronal firing and membrane potential dynamics in the somatosensory thalamus of awake mice. Cell Rep..

[bib47] Welker W.I. (1964). Analysis of sniffing of the albino rat. Behaviour.

[bib48] Yamashita T., Petersen C.C.H. (2016). Target-specific membrane potential dynamics of neocortical projection neurons during goal-directed behavior. eLife.

[bib49] Yamashita T., Pala A., Pedrido L., Kremer Y., Welker E., Petersen C.C.H. (2013). Membrane potential dynamics of neocortical projection neurons driving target-specific signals. Neuron.

[bib50] Zagha E., Ge X., McCormick D.A. (2015). Competing neural ensembles in motor cortex gate goal-directed motor output. Neuron.

