# Supplementary material for: Movement Initiation Signals in Mouse Whisker Motor Cortex
Source: Neuron. 2016 Dec 21;92(6):1368–82. doi: 10.1016/j.neuron.2016.12.001 (PMC5196025; doi:10.1016/j.neuron.2016.12.001)
Supplement: Document S1. Supplemental Experimental Procedures, Figures S1–S6, and Tables S1–S6 [file mmc1.pdf]

Neuron, Volume *92*

Supplemental Information

Movement Initiation Signals

in Mouse Whisker Motor Cortex

Varun Sreenivasan, Vahid Esmaeili, Taro Kiritani, Katia Galan, Sylvain Crochet, and Carl C.H. Petersen

Supplemental Information

Movement initiation signals in mouse whisker motor cortex

Varun Sreenivasan, Vahid Esmaeili, Taro Kiritani,
Katia Galan, Sylvain Crochet and Carl Petersen

Supplemental Information consists of:

Supplemental Figure S1 – related to Figure 1
Supplemental Figure S2 – related to Figure 2
Supplemental Figure S3 – related to Figure 3
Supplemental Figure S4 – related to Figure 4
Supplemental Figure S5 – related to Figure 5
Supplemental Figure S6 – related to Figure 6

Supplemental Table S1 – related to Figure 1
Supplemental Table S2 – related to Figure 2
Supplemental Table S3 – related to Figure 3
Supplemental Table S4 – related to Figure 4
Supplemental Table S5 – related to Figure 5
Supplemental Table S6 – related to Figure 6

Supplemental Experimental Procedures

Supplemental References

Supplemental Figure S1 (related to Figure 1)

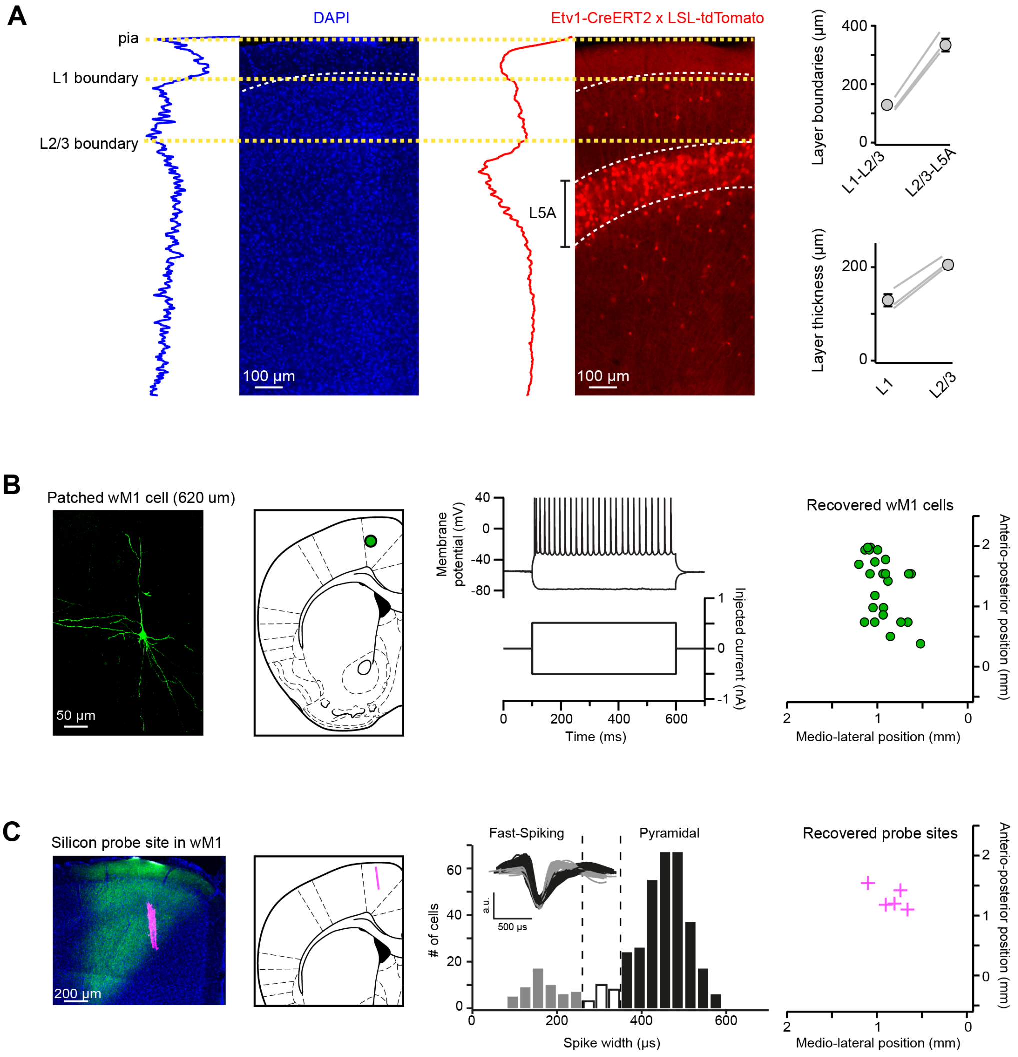

Supplemental Figure S1. **(A)** Coronal section of wM1 from an Etv1-CreERT2 x LSL-tdTomato mouse stained with DAPI (*left*) and expressing tdTomato in layer 5A (*middle*). Quantification of layer boundaries and layer thickness across 3 mice (*right*). Gray lines indicate individual mice. Circles with error bars represent group average as mean ± SEM. **(B)** Example layer 5 neuron that was recovered following in vivo whole-cell recording and its location on the Paxinos and Franklin (2001) mouse brain atlas (*left*). V_m response of the same cell upon injecting 500 pA of depolarizing and hyperpolarizing current (*middle*). Locations of all recovered cells in wM1 with respect to Bregma (*right*). **(C)** Coronal section through wM1 of an Emx1-Cre mouse expressing ChR2 in wS1 (*left*). Note the wS1 axons in wM1 (green) and the overlapping tract of the silicon probe, which was coated with DiI (pink). Histogram of spike

widths (quantified as peak to baseline interval) for all recorded single units (*middle*). The dotted lines represent the thresholds for classifying units as fast-spiking or pyramidal. Inset shows overlaid average spike waveform for all fast-spiking putative GABAergic (gray, n = 40) and pyramidal units (black, n = 213). Locations of all recovered silicon probe recording sites as estimated from DiI fluorescence (*right*).

Supplemental Figure S2 (related to Figure 2)

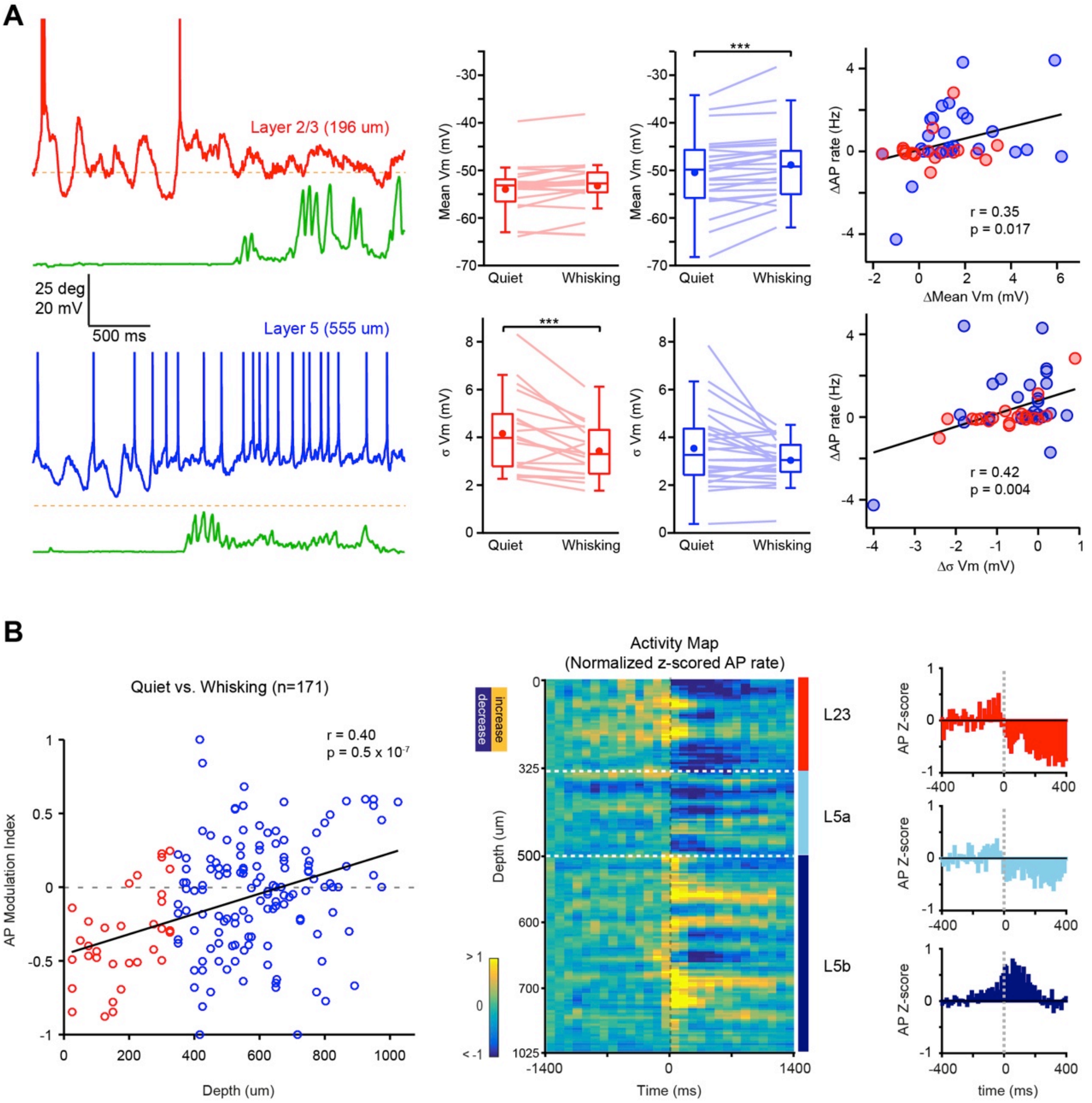

Supplemental Figure S2. (A) Example V_m recording from a layer 2/3 neuron (red) and a layer 5 neuron (blue), together with the corresponding whisker movement traces (green) (*left*). "Quiet" epochs were taken as 0.5 s preceding movement initiation and "Whisk" epochs were taken as 0.5 s following movement initiation. While significant changes in V_m, compared to baseline, occur during distinct phases of movement (Figure 2), the mean V_m, when computed over the entire 0.5 s of whisking and compared to the preceding 0.5 s of quiet wakefulness, does not change in layer 2/3 but depolarizes significantly in layer 5 (*middle*). The large V_m fluctuations (measured as the standard deviation of the V_m) evident during quiet wakefulness are significantly suppressed in layer 2/3. In layer 5, this suppression was evident

in some cells but was not significant across the population of all recorded layer 5 neurons (*middle*). Scatter plot of change in AP rate, measured during the whole-cell recording, comparing Whisking and Quiet (ΔAP rate) vs change in mean V_m (ΔMean V_m) or change in the standard deviation of the V_m (Δσ V_m) for all layer 2/3 (red circles) and layer 5 (blue circles) cells (*right*). ΔAP is correlated with both parameters, indicating that changes in AP firing rate depend not only on the V_m, but also on the size of the V_m fluctuations. **(B)** The AP modulation index, comparing Quiet and Whisking, positively correlates with the depth of the unit (*left*). Activity map (normalized z-scored AP rate) of all wM1 units (for a subset of trials of Figure 2) for whisking bouts when the mouse was quiet for at least 1.5 seconds and the whisking lasted longer than 1.5 seconds (*middle*). Note the transient change in firing rate preceding and following whisking onset. White dotted lines represent layer boundaries. Splitting the layer 5 units into units residing in 5A (light blue) and 5B (dark blue) shows that the increase in AP rates during the Pre-movement and Movement-onset periods are more prominent in layer 5B, while layer 5A behaves in a manner more similar to layer 2/3.

Supplemental Figure S3 (related to Figure 3)

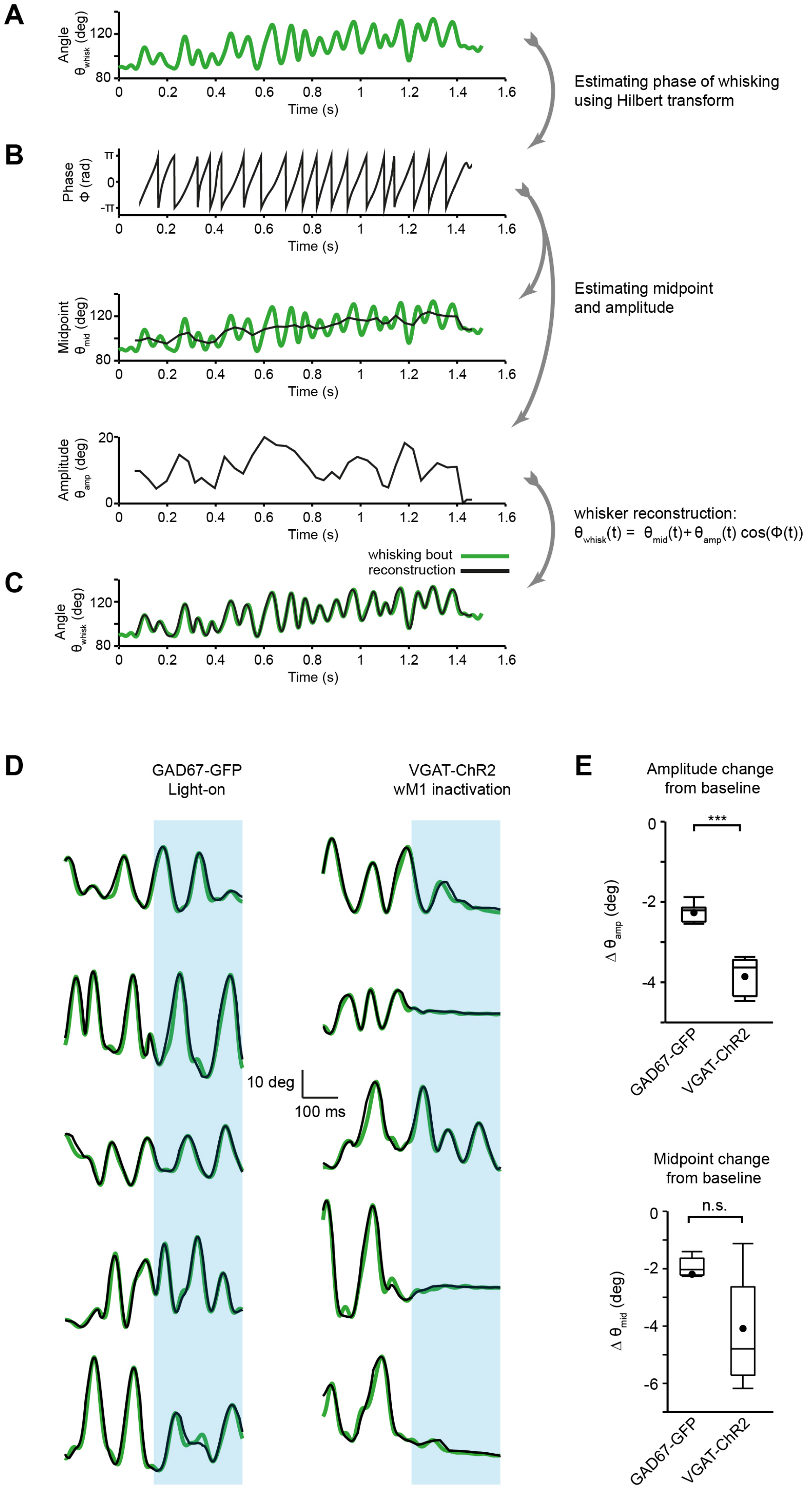

Supplemental Figure S3. (A) Example bout of rhythmic whisking. **(B)** Whisking was decomposed into its three constituent variables using the

Hilbert transform. These are the instantaneous phase (*top*), midpoint (*middle*) and amplitude (*bottom*). **(C)** Original whisker position (green) was compared to the reconstructed whisker position (black) using the three variables to check the accuracy of parameter estimation. Note the negligible difference between the two traces. (**D**) Example bouts of rhythmic whisking during control light applied on GAD67-GFP mice (*left*) and wM1 inactivation in VGAT-ChR2 mice (*right*). Green traces indicate the recorded whisking bouts and the overlaid black traces indicate the reconstruction following the procedure outlined in panels A-C. Note the reduction in amplitude of whisking and, in some cases, the complete halt of whisking, upon wM1 inactivation. (**E**) Quantification of the change in whisking amplitudes and midpoints comparing baseline (the 250 ms before light onset) and during light application (the 250 ms immediately after light onset) in GAD67-GFP (n = 7 mice) and wM1 of VGAT-ChR2 (n = 7 mice) animals. The change in amplitude ($\Delta\theta_{amp}$) is significantly more negative for VGAT-ChR2 mice compared to GAD67-GFP mice, indicating that wM1 inactivation tended to reduce whisking amplitude or stop whisking on a larger proportion of trials. The change in midpoint ($\Delta\theta_{mid}$) is not significantly different for VGAT-ChR2 mice compared to GAD67-GFP mice.

Supplemental Figure S4 (related to Figure 4)

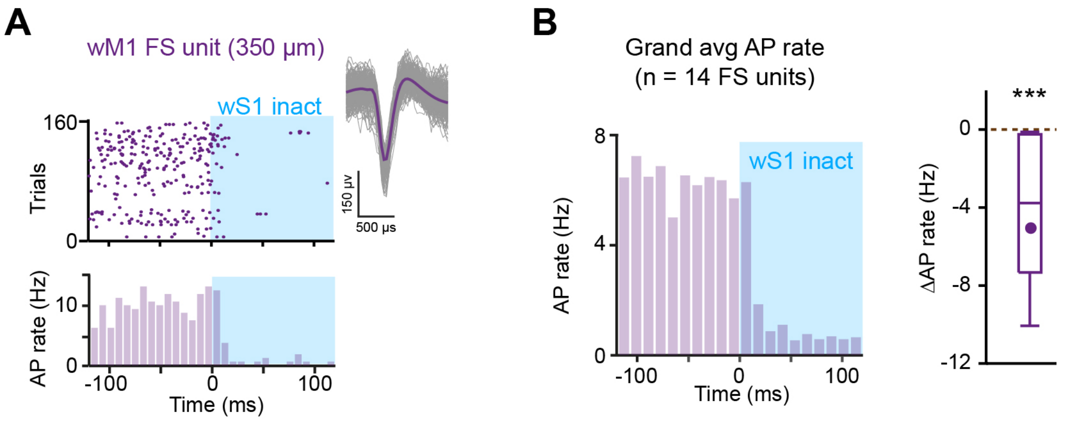

Supplemental Figure S4. (A) Example raster plot and PSTH of a wM1 fast-spiking putative GABAergic unit recorded in a VGAT-ChR2 mouse upon wS1 inactivation. Inset shows overlaid spikes (gray) from this unit together with the average spike waveform (purple). **(B)** The grand average AP rate, across 14 wM1 fast-spiking units, shows a decrease in activity when wS1 is inactivated. Quantification of change in AP rate, for all fast-spiking units, upon wS1 inactivation.

Supplemental Figure S5 (related to Figure 5)

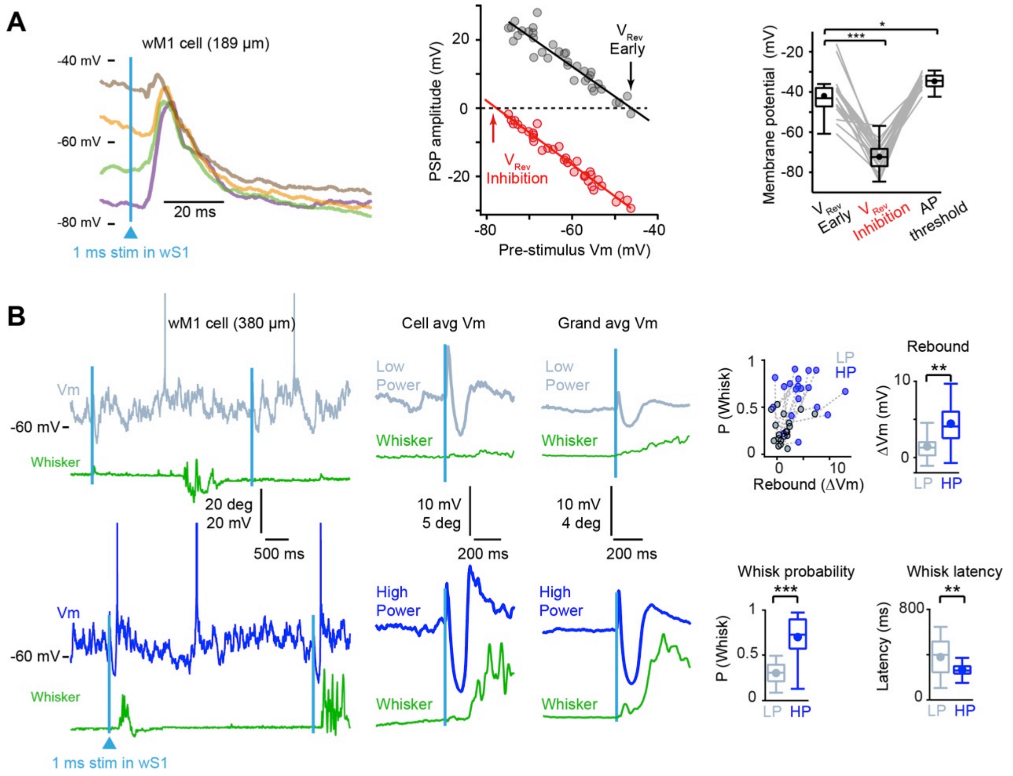

Supplemental Figure S5. (A) Example V_m traces from a layer 2/3 neuron in wM1 showing the dependence of the “Early” and “Inhibition” response amplitudes on the pre-stimulus V_m (*left*). Plot of the PSP amplitude vs the pre-stimulus V_m for the same cell (*middle*). The reversal potential of either response is the pre-stimulus V_m at which the response amplitude is 0 mV. Quantification of the “Early” and “Inhibition” reversal potentials and their comparison to the AP threshold (*right*). **(B)** Example V_m traces from a wM1 cell upon wS1 stimulation with low (gray, 2 mW) and high (blue, 9 mW) LED powers (*left*). The corresponding whisker traces (green) are shown below each V_m trace. Note the higher probability of whisking initiation, P(Whisk), following the stronger light stimulus. Average V_m and whisker traces for the same wM1 cell and grand average V_m and whisker traces across 19 wM1 cells for low (gray) and high (blue) powers (*middle*). Plot of P(Whisk) vs the size of the Rebound V_m for 19 wM1 cells (*right*). Each connected pair is one cell with the corresponding values of P(Whisk) and Rebound ΔV_m for low and high powers. Quantified across all cells, the stronger stimulus led to a larger Rebound ΔV_m along with a significantly higher P(Whisk) at significantly lower latencies (*right*).

Supplemental Figure S6 (related to Figure 6)

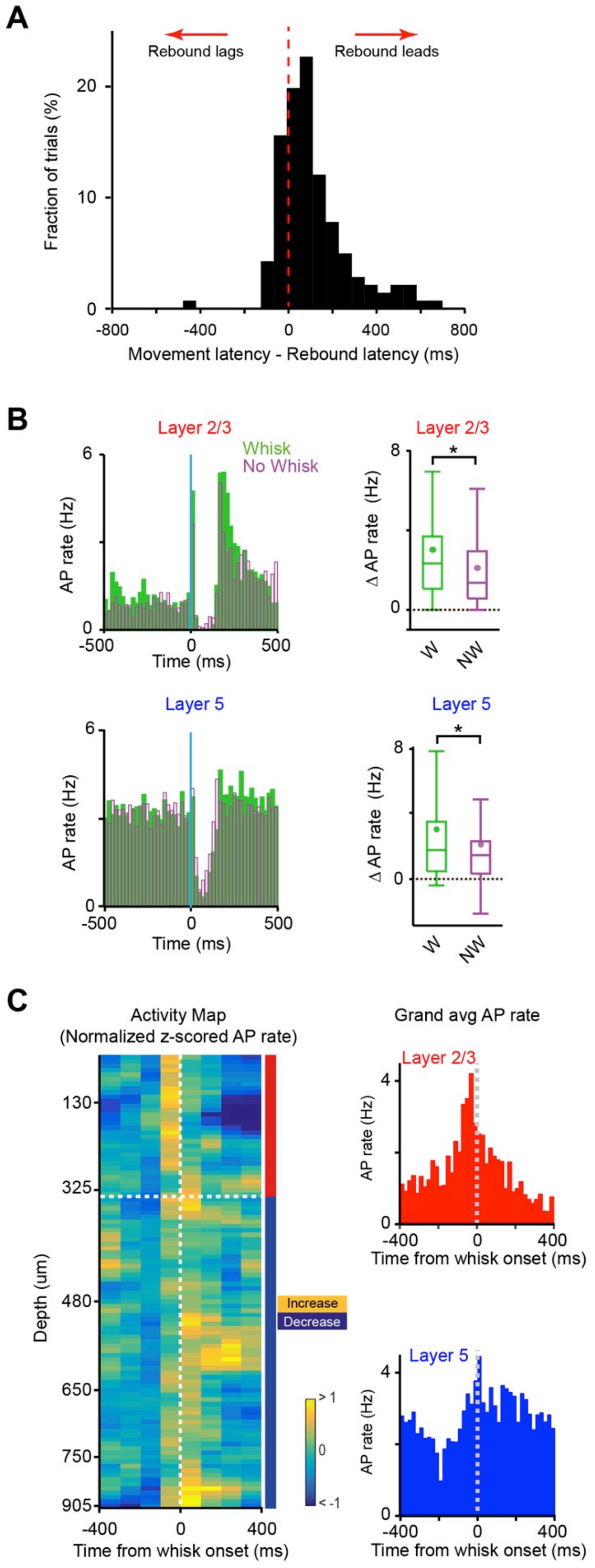

Supplemental Figure S6. (A) Histogram of the difference in latencies between evoked whisker movement and Rebound depolarization. All "Whisk"

trials from 19 whole-cell recordings were pooled to obtain the distribution. Note the bias of the distribution towards positive values, indicating that the Rebound depolarization precedes the whisker movement on the majority of trials **(B)** Grand average PSTHs of extracellular recordings following wS1 stimulation, for Whisk (magenta) and No Whisk (green) conditions, in layer 2/3 and layer 5 (*left*). Quantified across the population, the increase in AP firing rate from the Inhibition phase to the Rebound phase was significantly larger during Whisk trials compared to No Whisk trials in both L2/3 and L5 (*right*). **(C)** Laminar map of spiking activity for whisk trials (*left*). The z-scored PSTHs of individual units (100 ms bin size) were aligned to whisking onset and sorted according to their depth. A smoothing window (with size of 5 units) was applied across depth to obtain the smooth activity map. Grand average AP rates in layer 2/3 and layer 5 (*right*), for Whisk trials, aligned to whisking onset. Note the increase in AP firing rates, preceding movement onset, similar to self-initiated whisking in Figure 2.

Supplemental Table S1 (related to Figure 1)

	AP axis mean	AP axis s.e.m	ML axis mean	ML axis s.e.m	N
**Location of wS1 axons in frontal cortex**	1.15 mm	0.1 mm	0.87 mm	0.04 mm	4 mice

wM1 activation in Emx1-Cre	Median	IQR	Mean	N
**P(Whisk)**	1	0	0.97	6 mice
**Latency**	25 ms	8 ms	25.33 ms	6 mice
**Angle**	8.47 deg	10.14 deg	10.79 deg	6 mice
**FFT power**	43 deg^2	99.17 deg^2	72.62 deg^2	6 mice

	Median	IQR	Mean	P value	Test	N
**P(Whisk) Catch trials**	0.49	0.14	0.50	0.015	Wilcoxon	7 mice
**P(Whisk) wM1 inactivation**	0.26	0.16	0.31			

Supplemental Table S2 (related to Figure 2)

V_m	Median	IQR	Mean	n	N
L2/3 Baseline (B) V_m	-53.15 mV	5.08 mV	-54.15 mV	20 cells	16 mice
L2/3 Pre-Movement (P) V_m	-53.04 mV	5.26 mV	-53.66 mV	20 cells	16 mice
L2/3 Movement start (M) V_m	-53.67 mV	4.81 mV	-54.37 mV	20 cells	16 mice
L2/3 Late (L) V_m	-52.23 mV	4.54 mV	-52.93 mV	20 cells	16 mice
L5 Baseline (B) V_m	-49.45 mV	9.42 mV	-50.32 mV	26 cells	23 mice
L5 Pre-Movement (P) V_m	-49.14 mV	10.79 mV	-49.61 mV	26 cells	23 mice
L5 Movement start (M) V_m	-49.01 mV	10.02 mV	-48.97 mV	26 cells	23 mice
L5 Late (L) V_m	-48.12 mV	9.40 mV	-48.36 mV	26 cells	23 mice

AP rate	Median	IQR	Mean	n	N
L2/3 Baseline (B) AP rate	1.25 Hz	1.67 Hz	1.65 Hz	37 units	5 mice
L2/3 Pre-Movement (P) AP rate	1.16 Hz	1.68 Hz	1.80 Hz	37 units	5 mice
L2/3 Movement start (M) AP rate	0.61 Hz	1.26 Hz	1.28 Hz	37 units	5 mice
L2/3 Late (L) AP rate	0.38 Hz	1.17 Hz	0.94 Hz	37 units	5 mice
L5 Baseline (B) AP rate	1.72 Hz	3.61 Hz	2.69 Hz	134 units	5 mice
L5 Pre-Movement (P) AP rate	1.90 Hz	3.10 Hz	3.10 Hz	134 units	5 mice
L5 Movement start (M) AP rate	1.59 Hz	3.52 Hz	3.74 Hz	134 units	5 mice
L5 Late (L) rate	1.20 Hz	2.94 Hz	3.0 Hz	134 units	5 mice

ΔV_m	Median	IQR	Mean	P value	Test	n	N
L2/3 P - B	0.35 mV	1.07 mV	0.48 mV	0.09	Wilcoxon	20 cells	16 mice
L2/3 M - P	-0.62 mV	1.11 mV	-0.70 mV	0.01	Wilcoxon	20 cells	16 mice
L2/3 M - B	-0.26 mV	1.62 mV	-0.21 mV	0.37	Wilcoxon	20 cells	16 mice
L2/3 L - B	1.14 mV	2.66 mV	1.21 mV	0.02	Wilcoxon	20 cells	16 mice
L5 P - B	0.26 mV	1.35 mV	0.70 mV	0.02	Wilcoxon	26 cells	23 mice
L5 M - P	0.46 mV	1.16 mV	0.64 mV	0.07	Wilcoxon	26 cells	23 mice
L5 M - B	0.77 mV	2.42 mV	1.34 mV	0.01	Wilcoxon	26 cells	23 mice
L5 L - B	1.67 mV	2.13 mV	1.95 mV	0.00004	Wilcoxon	26 cells	23 mice

ΔAP	Median	IQR	Mean	P value	Test	n	N
L2/3 P - B	0.14 Hz	0.59 Hz	0.14 Hz	0.07	Wilcoxon	37 units	5 mice
L2/3 M - P	-0.38 Hz	0.56 Hz	-0.51 Hz	0.00004	Wilcoxon	37 units	5 mice
L2/3 M - B	-0.34 Hz	0.69 Hz	-0.37 Hz	0.001	Wilcoxon	37 units	5 mice
L2/3 L - B	-0.50 Hz	1.05 Hz	-0.70 Hz	0.0002	Wilcoxon	37 units	5 mice
L5 P - B	0.18 Hz	0.86 Hz	0.41 Hz	0.0006	Wilcoxon	134 units	5 mice
L5 M - P	0.05 Hz	1.82 Hz	0.64 Hz	0.26	Wilcoxon	134 units	5 mice
L5 M - B	0.15 Hz	1.80 Hz	1.05 Hz	0.03	Wilcoxon	134 units	5 mice
L5 L - B	-0.08 Hz	1.17 Hz	0.30 Hz	0.40	Wilcoxon	134 units	5 mice

AP thresh.	Median	IQR	Mean	P value	Test	n	N
L2/3	-33.8 mV	7.1 mV	-34.5 mV	0.97	Mann-Whitney	14 cells	11 mice
L5	-35 mV	5.0 mV	-33.7 mV			19 cells	17 mice

Supplemental Table S3 (related to Figure 3)

<table>
<tr><th></th><th>Phase</th><th colspan="6">Midpoint</th><th colspan="6">Amplitude</th></tr>
<tr><td rowspan="2">L2/3 V_m</td><td rowspan="2">6/12 cells</td><td colspan="3">pos</td><td colspan="3">neg</td><td colspan="3">pos</td><td colspan="3">neg</td></tr>
<tr><td colspan="3">1/12 cells</td><td colspan="3">2/12 cells</td><td colspan="3">3/12 cells</td><td colspan="3">—</td></tr>
<tr><td rowspan="2">L5 V_m</td><td rowspan="2">3/14 cells</td><td colspan="3">pos</td><td colspan="3">neg</td><td colspan="3">pos</td><td colspan="3">neg</td></tr>
<tr><td colspan="3">1/14 cells</td><td colspan="3">5/14 cells</td><td colspan="3">—</td><td colspan="3">3/14 cells</td></tr>
<tr><td rowspan="2">L2/3 AP rate</td><td rowspan="2">5/17 units</td><td colspan="2">pos</td><td colspan="2">neg</td><td colspan="2">NM</td><td colspan="2">pos</td><td colspan="2">neg</td><td colspan="2">NM</td></tr>
<tr><td colspan="2">—</td><td colspan="2">—</td><td colspan="2">4/17 units</td><td colspan="2">—</td><td colspan="2">—</td><td colspan="2">4/17 units</td></tr>
<tr><td rowspan="2">L5 AP rate</td><td rowspan="2">12/90 units</td><td colspan="2">pos</td><td colspan="2">neg</td><td colspan="2">NM</td><td colspan="2">pos</td><td colspan="2">neg</td><td colspan="2">NM</td></tr>
<tr><td colspan="2">16/90 units</td><td colspan="2">10/90 units</td><td colspan="2">15/90 units</td><td colspan="2">9/90 units</td><td colspan="2">10/90 units</td><td colspan="2">13/90 units</td></tr>
</table>

Data for Figure S3E

	Mouse	Median	IQR	Mean	P value	Test	N
**ΔAmp**	GAD67	-2.2 deg	0.35 deg	-2.2 deg	0.0005	Mann-Whitney	7 mice
	VGAT	-3.6 deg	0.90 deg	-3.8 deg			7 mice
**ΔMid**	GAD67	-2.0 deg	0.58 deg	-2.2 deg	0.053	Mann-Whitney	7 mice
	VGAT	-4.8 deg	3.0 deg	-4.0 deg			7 mice

Supplemental Table S4 (related to Figure 4)

**wS1 inactivation / Control**	**Median**	**IQR**	**Mean**	**P value**	**Test**	**N**
P (Whisk) in VGAT-ChR2	0.17	0.12	0.18	0.0003	Mann-Whitney	8 mice
P (Whisk) in GAD67-GFP	0.51	0.11	0.54			7 mice

	**Median**	**IQR**	**Mean**	**n**	**N**
**Latency to hyperpolarization**	10.3 ms	2.4 ms	10.73 ms	14 cells	8 mice

**Δ V_m / AP rate**	**Median**	**IQR**	**Mean**	**n**	**N**
L2/3 ΔV_m	-8.06 mV	3.69 mV	-8.12 mV	10 cells	6 mice
L5 ΔV_m	-11.35 mV	3.39 mV	-12.05 mV	4 cells	3 mice
L2/3 ΔAP rate	-0.24 Hz	1.01 Hz	-0.85 Hz	27 units	3 mice
L5 ΔAP rate	-0.86 Hz	1.79 Hz	-1.75 Hz	59 units	3 mice

	**P value**	**Test**
**ΔV_m L2/3 vs L5**	0.075	Mann-Whitney
**ΔAP rate L2/3 vs L5**	0.009	Mann-Whitney

Supplemental Table S5 (related to Figure 5)

wS1 activation in Emx1-Cre	Median	IQR	Mean	N
P(Whisk)	0.72	0.32	0.69	15 mice
Latency	260 ms	65.27 ms	266.64 ms	15 mice

Vm	Median	IQR	Mean	n	N
L2/3 Early ΔV_m	12.14 mV	3.53 mV	12.11 mV	10 cells	8 mice
L2/3 Inhibition ΔV_m	-10.50 mV	6.13 mV	-10.08 mV	10 cells	8 mice
L2/3 Rebound ΔV_m	4.32 mV	4.21 mV	5.79 mV	10 cells	8 mice
L5 Early ΔV_m	7.11 mV	9.06 mV	5.97 mV	9 cells	6 mice
L5 Inhibition ΔV_m	-10.72 mV	7.35 mV	-12.25 mV	9 cells	6 mice
L5 Rebound ΔV_m	3.61 mV	3.69 mV	2.93 mV	9 cells	6 mice

AP rate	Median	IQR	Mean	n	N
L2/3 Early ΔAP rate	0.48 Hz	3.80 Hz	2.98 Hz	36 units	3 mice
L2/3 Inhibition ΔAP rate	-0.92 Hz	1.02 Hz	-1.01 Hz	36 units	3 mice
L2/3 Rebound ΔAP rate	0.80 Hz	2.00 Hz	1.98 Hz	36 units	3 mice
L5 Early ΔAP rate	-0.84 Hz	2.44 Hz	-1.12 Hz	66 units	3 mice
L5 Inhibition ΔAP rate	-1.54 Hz	3.54 Hz	-2.45 Hz	66 units	3 mice
L5 Rebound ΔAP rate	0.13 Hz	2.09 Hz	0.58 Hz	66 units	3 mice

	P value	Test
Early ΔV_m L2/3 vs L5	0.017	Mann-Whitney
Inhibition ΔV_m L2/3 vs L5	0.49	Mann-Whitney
Rebound ΔV_m L2/3 vs L5	0.11	Mann-Whitney
Early ΔAP rate L2/3 vs L5	0.00001	Mann-Whitney
Inhibition ΔAP rate L2/3 vs L5	0.014	Mann-Whitney
Rebound ΔAP rate L2/3 vs L5	0.01	Mann-Whitney

V_{rev} Early *vs* AP threshold	Median	IQR	Mean	P value	Test	n	N
V_{rev} Early	-43.1 mV	9.2 mV	-42.0 mV	0.015	Wilcoxon	18 cells	12 mice
AP threshold	-34.4 mV	5.3 mV	-34.7 mV				

Supplemental Table S6 (related to Figure 6)

Modulation Index	Pearson r	P value	Test	n	N
L2/3	-0.239	0.249	Permutation	25 units	2 mice
L5	0.421	0.017	Permutation	32 units	2 mice

**wS1 activation / wM1 inactivation in Emx1-Cre**	Median	IQR	Mean	N
P(Whisk) before Muscimol	0.98	0.12	0.93	8 mice
P(Whisk) after Muscimol	0.25	0.18	0.24	8 mice
P(Whisk) before Ringer's	0.95	0.15	0.92	7 mice
P(Whisk) after Ringer's	0.93	0.23	0.86	7 mice

P(Whisk)	P value	Test
Before *vs* After Muscimol	0.008	Wilcoxon
Before *vs* After Ringer's	0.22	Wilcoxon

Supplemental Experimental Procedures

All experiments were carried out in accordance with protocols approved by the Swiss Federal Veterinary Office.

Implantation of metal headpost

Adult (6-9 week old) male and female mice were deeply anesthetized with isoflurane (3% with O_2) and held in a nose-clamp. Carprofene (0.5 mg/ml, 300 µl, i.p.) was administered before the surgery. During surgery, the isoflurane concentration was held between 1-2%. The body temperature was maintained at 37°C by a heating pad. An ocular ointment (Viscotears, Alcon) was applied over the eyes to prevent drying. A mixture of 2% Lidocaine (1:10 dilution) and 0.5% Bupivacaine (1:2 dilution) was injected subcutaneously. The scalp was cut open to expose the skull. The periosteal tissue, covering the scalp, was removed by gently scraping with a scalpel. The skull was then cleaned with Betadine. A light-weight metal head-post was fixed to the right hemisphere with cyano-acrylate glue (Henkel, Dusseldorf, Germany). A thin layer of glue was also applied over the left hemisphere to protect the skull. The glue was allowed to dry for 10 minutes. A chamber was made by building a wall with dental cement (Paladur, Heraeus Kulzer, Hanau, Germany) along the edge of the bone covering the left hemisphere. Dental cement was also used on the right hemisphere to reinforce the attachment of the head-post. The animal was returned to its home cage and Ibuprofen was added to the drinking water for 3 days following surgery.

Intrinsic optical signal imaging

Intrinsic optical signal imaging (Grinvald et al., 1986) was carried out to map the location of the C2 barrel column in primary whisker somatosensory cortex (wS1). All whiskers except the C2 whisker on either side were trimmed. The animal was anesthetized and head-fixed in a custom made metal frame. After induction of anesthesia, isoflurane concentration was maintained between 1-1.5%. Body temperature was maintained at 37°C. The recording chamber, over the left hemisphere, was filled with warm Ringer's solution (in mM: 135 NaCl, 5 KCl, 1 $MgCl_2$, 1.8 $CaCl_2$, 5 HEPES; pH 7.4) and covered with a small

glass cover slip. A reference image of the surface vasculature was first obtained under green illumination (525 nm). The contralateral C2 whisker was then deflected with a glass capillary attached to a piezo actuator (PICMA, PI Ceramic) and the resulting intrinsic signal response was imaged under 630 nm illumination by a CMOS camera (Photon Focus, Lachen, Switzerland) through a stereomicroscope (Leica, Wetzlar, Germany). Stimulation and blank trials were interspersed. Each trial consisted of 4 s of baseline followed by 4 s of whisker stimulation at 10 Hz (or blank). The inter-trial interval was 30 s. The fractional change in reflectance was calculated by subtracting the average baseline reflectance ($R_{Baseline}$) from the average reflectance during the stimulation period (R_{Stim}) and dividing the resulting value by the average baseline reflectance: (R_{Stim}-$R_{Baseline}$) / $R_{Baseline}$. Acquisition and processing of the images was done with custom routines written in LabView (National Instruments, Austin TX, USA) and Matlab (Natick, MA, USA).

Virus injections

Two viruses were used in this study. An AAV2/5 virus expressing double-floxed humanized ChR2 (histidine 134 converted to arginine) fused to EYFP under the control of the EF1α promoter (AAV2/5.DIO.EF1α. hChR2(H134R).EYFP virus made by Penn Vector Core, Philadelphia, PA, USA) was used to express Channelrhodopsin-2 (ChR2), in wM1 (Figure 1) or wS1 (Figures 5 and 6) of Emx1-Cre mice (RRID IMSR_JAX:005628), to selectively express ChR2 in excitatory pyramidal cells (Chan et al., 2001; Gorski et al., 2002). A second AAV2/9 virus expressing double-floxed tdTomato under the control of the CAG promoter (AAV2/9.FLEX.CAG.tdTomato virus made by Penn Vector Core, Philadelphia, PA) was used to anatomically map the wS1 inputs to wM1 in Emx1-Cre mice (Figure 1). wS1 injections were targeted to the C2 barrel column (identified through intrinsic optical signal imaging). A small craniotomy (< 0.5 mm in diameter) was made over wS1. The dura was left intact. An injection pipette (internal tip diameter ~20 μm) was tip-filled with the virus solution and lowered into the brain. Injections were carried out at two different depths (300 μm and 800 μm below the pia) to infect cells in both supra and infra-granular layers of wS1 cortex. For the anatomical labeling, 30 nl of virus was injected at each

depth. For the ChR2 experiments, 250 nl of virus was injected at each depth. wM1 injections were carried out similarly but targeted through stereotactic coordinates to 1 mm anterior and 1 mm lateral to Bregma. The pipette was allowed to remain in the brain for 5 minutes before being retracted slowly over a period of 8-10 minutes to prevent backflow of the virus along the shaft. The craniotomy was covered with a silicone elastomer (Kwik-Cast, World Precision Instruments, Sarasota, FL, USA) and a layer of dental cement was added over the elastomer, to prevent the animal from taking it off in the cage. The virus was allowed to express for 4 weeks.

Habituation to head fixation

Animals were adapted to head-restraint over 3 days in sessions of increasing duration for periods of 20, 40 and 60 minutes respectively. Training began 4 days after implantation for wild-type mice. For mice where ChR2 virus was injected, training began 4 weeks after virus injection. Ambient blue light was present all throughout the training phase and also on the day of the experiment. Blue background illumination provided lighting to film the whiskers as well as masking the optogenetic stimulus. At the end of each session, the mice were rewarded with fruit juice.

Whisker filming

Whisker movements were filmed at 500 Hz with a high-speed camera (CL 600X2/M, Optronis, Kehl, Germany). The exposure time was 1 ms. All whiskers were trimmed except the C2 whiskers on either side. Filming was carried out in blocks of 30-60 s. Whisker angle was quantified using custom routines implemented in IgorPro (Wavemetrics, Lake Oswego, OR, USA).

Optogenetics

A fiber-coupled high power blue LED system (Thorlabs, Newton, NJ, USA) was used to stimulate ChR2 expressed in wS1. A 400μm (NA 0.39) fiber optic cable (M28L05, Thorlabs) was coupled to a 470 nm high power LED (M470F1, Thorlabs). The bone surrounding the injection craniotomy in wS1 or wM1 was thinned until the underlying vasculature was clearly visible. The fiber optic cable was lowered until the tip touched the bone surface. Stimuli varied

depending on the experiment. Optogenetic activation of wM1 (Figure 1) made use of 25 pulses (pulse duration of 5 ms) delivered at 50 Hz. Optogenetic inactivation of wM1 (Figure 1) and wS1 (Figure 4) made use of a single blue light pulse delivered for 1 sec. Optogenetic activation of wS1 made use of a single pulse delivered for 1 ms (Figure 5 and Figure 6). The peak light power was either 9 mW (high power) or 2 mW (low power). The inter-pulse interval was randomly chosen between 3 and 9 seconds by custom routines implemented in LabView (National Instruments, Austin TX, USA) and Matlab (Natick, MA, USA). As a control we applied the same light stimuli to GAD67-GFP mice (Tamamaki et al., 2003).

Electrophysiology

In vivo whole-cell recordings

"Blind" *in vivo* whole-cell recordings (Margrie et al., 2002) were carried out in whisker motor cortex (wM1) of awake head-restrained animals. In most experiments, the recordings were targeted to 1 mm anterior and 1 mm lateral to Bregma. The membrane potential of 70 neurons (N = 51 mice) in wM1 was recorded. Of these, 19 neurons were recorded in Emx1-Cre mice (N = 12 mice; RRID IMSR_JAX:005628), 6 neurons in VGAT-ChR2 mice (N = 3 mice; RRID IMSR_JAX:014548) (Zhao et al., 2011), 8 neurons in PV-Cre x Rosa-LSL-ChR2 mice (N = 6 mice) (PV-Cre mice: Hippenmeyer et al., 2005, RRID IMSR_JAX:008069; Rosa-LSL-ChR2 mice: Madisen et al., 2012, RRID IMSR_JAX:012569) and 37 neurons in C57BL/6J mice (N = 30 mice). A small craniotomy (< 300 μm) was made over wM1 and the dura was removed. The pipette internal solution contained 135 mM potassium gluconate, 4 mM KCl, 10 mM sodium phosphocreatinine, 4 mM MgATP, 0.3 Na_3GTP, 10 mM HEPES (pH 7.3). Biocytin (Biotium, Fremont, CA, USA) was added to the internal solution to give a final concentration of 2-4 mg/ml. An Ag/AgCl wire, attached to the head-stage, was used as the recording electrode. Patch pipettes were pulled from borosilicate glass capillaries and had a tip-resistance of 4-8 MΩ. The pipettes were filled with internal solution, fixed onto the head-stage and lowered into the recording chamber (built with dental cement), which was filled with Ringer's solution ("bath solution"). A second Ag/AgCl electrode connected to the head-stage was dipped into the recording

chamber and used as the reference electrode. Positive pressure (180 millibars) was applied and the pipette was lowered until 150 μm below the pial surface. The pressure was then reduced to 18-25 millibars. The pipette was advanced in 2 μm steps. On encountering a cell, negative pressure was applied to allow the formation of a gigaohm seal. Once seal formation was achieved, brief pulses of suction were applied to break into the cell. All recordings were carried out in current clamp mode. At the beginning of every recording, depolarizing and hyperpolarizing current pulses were injected to ensure that all cells fired action potentials upon positive current injection. The membrane potential was sampled at 20 kHz in blocks of 30 to 60 seconds. Signals were digitized and recorded on an ITC-18 (Instrutech, Longmont, CO, USA) analog to digital converter board, using custom routines implemented in IgorPro (Wavemetrics, Lake Oswego, OR, USA). Liquid junction potential was not corrected.

Silicon probe recordings

Extracellular spikes were recorded using a silicon probe (A1x32-Poly2-10mm-50s-177, NeuroNexus, MI, USA) with 32 recording sites along a single shank covering 775 μm of the cortical depth. The back of the probe was coated with DiI for post-hoc recovery of the recording location. The probe was lowered gradually until the tip was positioned at a depth of ~1000 μm under the wM1 pial surface. The neural data were filtered between 0.3 Hz and 7.5 kHz and amplified using a digital headstage (CerePlex™ M32, Blackrock Microsystems, UT, USA). The headstage digitized the data with a sampling frequency of 30 kHz. The digitized signal was transferred to our data acquisition system (CerePlex™ Direct, Blackrock Microsystems, UT, USA) and stored on an internal HDD of the host PC for offline analysis. Spiking activity on each probe was detected and sorted into different clusters using KlustaSuite, an open source spike sorting software suited for dense multi-electrode recordings (Rossant et al., 2016). After an automated clustering step, clusters were manually sorted and refined. Only well isolated single units were included in the data set.

Histology and cell locations

At the end of the recording session, mice were perfused with 4% PFA in PBS. The brain was post-fixed overnight in PFA at room temperature. 80 µm thick serial sections of the frontal cortex were cut on a vibratome (VT 100; Leica, Wetzlar, Germany). The slices were incubated in blocking solution containing 5% normal goat serum and 0.3% Triton X for 1 hour. They were then transferred to the staining solution containing 0.3% Triton X and 1:2000 of Streptavidin conjugated to Alexa 488 or 647 (Life Technologies, Carlsbad, CA, USA). DAPI was used as a counterstain. The slices were then mounted and imaged under a slide scanner (Olympus VS120) and a confocal microscope (Zeiss LSM700). To determine the location of the neuron in frontal cortex, a widefield image of the entire slice containing the cell was acquired and compared to a mouse brain atlas (Paxinos and Franklin, 2001) to obtain anterio-posterior (AP) co-ordinates. Medio-lateral (ML) distance was measured from the cell body to the midline. Depth was measured vertically from the pial surface to the cell body. In the cases where the cell could not be recovered, the manipulator reading was taken as the depth. In the cases where the cell was recovered, the actual depth was measured on the slice.

Estimation of layer boundaries

Two methods were used to estimate layer boundaries in wM1. In DAPI stained sections of wM1, intensity plots across the entire cortical depth were calculated to check if there were any sharp changes in cell densities that might indicate layer boundaries. This method was useful in estimating the layer 1-layer 2/3 boundary but was not useful for estimating other layer boundaries. As a second method, we used the Etv1-CreERT2 (B6.Cg-Etv1$^{tm1.1\ (Cre/ERT2)Zjh/J}$) (Taniguchi et al., 2011; RRID IMSR_JAX:013048) x Rosa-LSL-tdTomato mice (B6;129S6-Gt(ROSA)26Sor$^{tm9(CAG-tdTomato)Hze}$/J) (Madisen et al., 2010; RRID IMSR_JAX:007908). 100 µl of a 20 mg/ml solution of Tamoxifen in corn-oil was administered intra-peritoneally for 3 consecutive days. The animals were sacrificed 9 days after the first tamoxifen injection. Histological analysis showed dense labeling of somata in layer 5A of wM1. Layer 1 was brightly labeled. Layer 2/3 was dark due to very few neurons expressing tdTomato. Layer 5A showed the strongest signal. This

served as a good approach to estimate the layer 1-layer 2/3 boundary as well as the layer 2/3-layer 5A boundary. Quantified across animals, the layer 1 boundary was 129 ± 13 μm (n = 3 mice) below the pia and the layer 2/3 boundary was 334 ± 22 μm (n = 3 mice) below the pia. The thickness of layer 1 was found to be 129 ± 13 μm (n = 3 mice) and the thickness of layer 2/3 was found to be 205 ± 9 μm (n = 3 mice). These layer boundary values have been used to separate the wM1 cells into layer 2/3 and layer 5 populations.

Muscimol inactivation

Inactivation of wM1 was carried out with 5 mM of the $GABA_A$ agonist, muscimol (BioTrend, Koln, Germany). A small craniotomy (< 300 μm) was made over wM1. The skull was covered with Kwik-Cast. Following surgery, the animal was returned to its home cage and allowed to recover for 2-3 hours. Control whisker movements, elicited by single 1 ms blue light flashes in wS1, were first obtained. The animal was then moved to the injection setup. 100 nl of muscimol (mixed with a small volume of Chicago Sky Blue) was injected at 900 μm, 700 μm, 500 μm, 300 μm and 100 μm each respectively, below the surface in wM1. The entire injection protocol was carried out over a period of 20 to 30 minutes. The animal was then moved back to the setup and 1 ms flashes of blue light were applied over wS1 to elicit whisker movements. The total time from the first injection of muscimol at 900 μm and re-testing was approximately 40 to 50 minutes.

Data analysis

Classification of units as fast-spiking or pyramidal

Units were classified as fast-spiking interneurons or putative pyramidal cells based on their mean spike waveform. Extracellular spike width was quantified as the peak-to-baseline interval. Single units with spike width < 0.26 ms were classified as fast-spiking (40 units) and units with width > 0.35 ms were classified as pyramidal (213 units). Units with intermediate spike width were excluded from the data set (14 units).

Movement onset detection

For assessing wM1 dynamics during self-initiated whisking, we analyzed epochs of whisking that lasted for at least 500 ms which were preceded by a quiet period of at least 500 ms. Movement onset times were estimated using custom routines written in Matlab. Briefly, movements lasting for 500 ms with absolute angles greater than 5 degrees above baseline (mean whisker position in the preceding 500 ms quiet period) were considered to be "Whisk" epochs. The movement onset time was taken as the time at which the absolute angle exceeded 1 degree above baseline. V_m traces and PSTHs of recorded cells were aligned to the onset of movement (t = 0 ms). Four phases were quantified: "Baseline" (B): -400 to -200 ms; "Pre-Movement" (P): -100 to 0 ms; "Movement start" (M): 0 to +100 ms; "Late" (L): +200 to +400 ms (Figure 2).

Activity map of self-initiated whisking

To obtain a laminar map of spiking activity across the entire depth of the recording, the PSTH of each unit was computed (100 ms bin size) around the whisking onset and then z-scored. The mean z-score value during the "Baseline" window (-400 to -200 ms) was subtracted from the z-score in each bin. The units were sorted according to their depth and a smoothing window (5 units wide) was applied across depth to obtain a visually smoother and continuous map.

Whisker decomposition

To decompose whisking bouts, we used the Hilbert transform approach of Hill et al. (2011). Whisker angular motion, θ, was broken down into three variables (amplitude $\theta_{amp}(t)$, midpoint $\theta_{mid}(t)$, and phase $\phi(t)$) at each time point. Whisking bouts that lasted more than half a second were selected and band-pass filtered (4-25 Hz). Instantaneous phase ($\phi(t)$) was computed using the Hilbert transform, where $\phi(t) = 0$ corresponds to the most protracted position of the whisk cycle and $\phi(t) = \pm\pi$ indicates the end of retraction. Whisking amplitude ($\theta_{amp}(t)$) at phase 0 and π was defined as:

$$\theta_{amp\,(\phi(t)=0\,\&\,\phi(t)=\pm\pi)} = (\theta_{\phi(t)=0} - \theta_{\phi(t)=\pm\pi})\,/2$$

Whisking amplitude at other times was computed by linearly interpolating between these points. Similarly whisking midpoint ($\theta_{mid}(t)$) was defined at phase 0 and π as:

$$\theta_{mid\,(\phi(t)=\,0\,\&\,\phi(t)=\,\pm\pi)} = \theta_{\phi(t)=\,\pm\pi} + (\theta_{\phi(t)=\,0} - \theta_{\phi(t)=\,\pm\pi})\,/2$$

Whisking midpoint at other times was computed by linearly interpolating between these points. Only those whisk cycles with amplitudes larger than 7.5 deg were considered for analysis, to exclude twitching and chattering.

AP rate tuning curves for whisking variables

We analyzed all units that had a mean AP firing rate of greater than 0.5 Hz during whisking. Tuning curves were computed by first binning the whisking variables (θ_{amp}, θ_{mid}, and ϕ) into 50 equally probable bins (2% percentiles). Next, a histogram of the firing rate was computed for each unit by counting the number of spikes in each bin divided by the amount of time spent in that bin. To test for significance, the distribution of the variable at all times (a uniform distribution because of equally probable binning) was compared to its distribution at spike times. P values were calculated using a 2-sample Kolmogorov-Smirnov test for amplitude and midpoint and a 2-sample Kuiper test for phase.

V_m correlation with whisking variables

The modulation of V_m by phase (ϕ) was determined by averaging the V_m traces of all whisk cycles aligned at phase zero (most protracted position of the whisk cycle). Modulation depth was computed as peak-to-trough of the average V_m trace. Significance was assessed by shuffling the individual V_m traces in a cyclic manner (circshift function in Matlab) and then computing the modulation depth on the average of these individual shuffled traces. This procedure was repeated 1000 times and the observed modulation depth was compared to the histogram of the shuffled modulation depths. The p value in this case is the fraction of shuffled modulation depths ≥ observed modulation depth

To quantify V_m modulation by the slow whisking variables, we first removed any trends in the V_m trace that might have been caused by drifts in

the recording over time. Individual recording blocks (20-60 sec) were concatenated to give one long continuous recording. This long recording was first divided into 5 sec blocks. The histograms of the V_m in blocks 4 to 14 were plotted. Typically, this histogram is bimodal since the V_m oscillates between a relatively depolarized state and a relatively hyperpolarized state. The hyperpolarized state values in blocks 4 to 14 were estimated at the first peak of this bimodal histogram and then averaged to give a single hyperpolarized state value. The V_m in each block was then offset so that the individual hyperpolarized state peak value for that histogram would coincide with the calculated average. We next computed mean V_m, mean θ_{amp} and mean θ_{mid} for each whisk cycle across all whisking bouts. Mean V_m values were plotted against the slow variables and the best linear fit to the data was calculated. To test for significance, we first resampled the data 1000 times (each time fitting a line to the resampled data) to obtain a resampled distribution of slopes. Next, we shuffled the values of the slow variable against the mean V_m on each whisk cycle 1000 times (each time fitting a line to the shuffled data) to obtain a shuffled distribution of slopes. We then compared the two distributions. The p value, in this case, is the probability of the shuffled values being ≥ the resampled distribution, for positive slopes, and being ≤ the resampled distribution, for negative slope.

Calculation of V_m response latencies

The magnitude of the V_m response in wM1, upon wS1 inactivation (Figure 4), was calculated by subtracting the mean V_m in 50 to 100 ms following stimulus onset from mean baseline V_m, measured during the -50 to 0 ms preceding stimulus onset. The latency of the response was taken as the time at which the change in V_m reached 5% of the response magnitude.

Activity map of Whisk / No Whisk difference

To obtain a laminar map of differences in AP firing rate for Whisk vs No Whisk trials following wS1 activation, the PSTH (100 ms bin size) of each unit was computed for Whisk and No Whisk conditions respectively. The No Whisk PSTH was subtracted from the Whisk PSTH and the resulting difference was z-scored. The mean z-score during baseline (-500 to 0 ms prestimulus time)

was computed and this value was subtracted from the z-score value in each bin. The units were sorted according to their depth and a smoothing window (5 units wide) was applied across depth to obtain a visually smoother and continuous map.

Modulation Index

Modulation index for wS1 evoked whisking was calculated using the following equation:

$$\mathrm{MI} = \frac{\text{Rebound AP rate (Whisk)} - \text{Rebound AP rate (No Whisk)}}{\text{Rebound AP rate (Whisk)} + \text{Rebound AP rate (No Whisk)}}$$

where the “Rebound” AP rate is the mean AP rate in 200 to 300 ms following the wS1 stimulus.

Modulation index for self-initiated whisking was calculated using the following equation:

$$\mathrm{MI} = \frac{\text{AP rate (Movement)} - \text{AP rate (Baseline)}}{\text{AP rate (Movement)} + \text{AP rate (Baseline)}}$$

where the “Movement” AP rate is the mean AP rate in 0 to 100 ms of the whisker movement onset aligned PSTH and “Baseline” AP rate is the mean AP rate in -400 to -200 ms of the whisker movement onset aligned PSTH.

Statistics

All group data are presented as boxplots. On each box, the central mark indicates the median, and the bottom and top edges of the box indicate the 25th and 75th percentiles, respectively. The whiskers extend to the most extreme data points not considered outliers. Outliers were considered as points lying at a distance of 1.5 x IQR above the 75th percentile or 1.5 x IQR under the 25th percentile, giving a coverage of ~99%. The mean is also indicated. Statistical testing was carried out in Matlab. All group comparisons were performed using Wilcoxon signed-rank test and Wilcoxon-Mann-Whitney test for paired and unpaired data respectively. Analysis of individual neurons was performed using non-parametric permutation tests.

Supplemental References

Chan, C.H., Godinho, L.N., Thomaidou, D., Tan, S.S., Gulisano, M., and Parnavelas, J.G. (2001). Emx1 is a marker for pyramidal neurons of the cerebral cortex. Cereb. Cortex *11*, 1191-1198.

Gorski, J.A., Talley, T., Qiu, M., Puelles, L., Rubenstein, J.L., and Jones, K.R. (2002). Cortical excitatory neurons and glia, but not GABAergic neurons are produced in the Emx1 expressing lineage. J. Neurosci. *22*, 6309-6314.

Grinvald, A., Lieke, E., Frostig, R.D., Gilbert, C.D., and Wiesel, T.N. (1986). Functional architecture of cortex revealed by optical imaging of intrinsic signals. Nature *324*, 361-364.

Hippenmeyer, S., Vrieseling, E., Sigrist, M., Portmann, T., Laengle, C., Ladle, D.R., and Arber, S. (2005). A developmental switch in the response of DRG neurons to ETS transcription factor signaling. PLoS Biol. *3*, e159.

Madisen, L., Zwingman, T.A., Sunkin, S.M., Oh, S.W., Zariwala, H.A., Gu, H., Ng, L.L., Palmiter, R.D., Hawrylycz, M.J., Jones, A.R., Lein, E.S., and Zeng, H. (2010). A robust and high-throughput Cre reporting and characterization system for the whole mouse brain. Nat. Neurosci. *13*, 133-140.

Madisen. L., Mao, T., Koch, H., Zhuo, J.M., Berenyi, A., Fujisawa, S., Hsu, Y.W., Garcia, A.J., Gu, X., Zanella, S., Kidney, J., Gu, H., Mao, Y., Hooks, B.M., Boyden, E.S., Buzsáki, G., Ramirez, J.M., Jones, A.R., Svoboda, K., Han, X., Turner, E.E., and Zeng, H. (2012). A toolbox of Cre-dependent optogenetic transgenic mice for light-induced activation and silencing. Nat. Neurosci. *15*, 793-802.

Margrie, T.W, Brecht, M., and Sakmann, B. (2002). In vivo low-resistance, whole-cell recordings from neurons in the anaesthetized and awake mammalian brain. Pflugers Arch. *444*, 491-498.

Paxinos, G., and Franklin, K. (2001). The mouse brain in stereotaxic coordinates. 2nd Edition. Academic Press, San Diego.

Tamamaki, N., Yanagawa, Y., Tomioka, R., Miyazaki, J., Obata, K., and Kaneko, T. (2003). Green fluorescent protein expression and colocalization with calretinin, parvalbumin, and somatostatin in the GAD67-GFP knock-in mouse. J. Comp. Neurol. *467*, 60-79.

Taniguchi, H., He, M., Wu, P., Kim, S., Paik, R., Sugino, K., Kvitsiani, D., Fu, Y., Lu, J., Lin, Y., Miyoshi, G., Shima, Y., Fishell, G., Nelson, S.B., and Huang, Z.J. (2011). A resource of Cre driver lines for genetic targeting of GABAergic neurons in cerebral cortex. Neuron *71*, 995-1013.

Zhao, S., Ting, J.T., Atallah, H.E., Qiu, L., Tan, J., Gloss, B., Augustine, G.J., Deisseroth, K., Luo, M., Graybiel, A.M., and Feng, G. (2011). Cell type–specific channelrhodopsin-2 transgenic mice for optogenetic dissection of neural circuitry function. Nat. Methods *8*, 745-752.
